# VRK1 as a synthetic lethal target in *VRK2* promoter–methylated cancers of the nervous system

**DOI:** 10.1172/jci.insight.158755

**Published:** 2022-10-10

**Authors:** Jonathan So, Nathaniel W. Mabe, Bernhard Englinger, Kin-Hoe Chow, Sydney M. Moyer, Smitha Yerrum, Maria C. Trissal, Joana G. Marques, Jason J. Kwon, Brian Shim, Sangita Pal, Eshini Panditharatna, Thomas Quinn, Daniel A. Schaefer, Daeun Jeong, David L. Mayhew, Justin Hwang, Rameen Beroukhim, Keith L. Ligon, Kimberly Stegmaier, Mariella G. Filbin, William C. Hahn

**Affiliations:** 1 Department of Medical Oncology, Dana-Farber Cancer Institute, Harvard Medical School, Boston, Massachusetts, USA.; 2 Broad Institute of MIT and Harvard, Cambridge, Massachusetts, USA.; 3 Department of Pediatric Oncology, Dana-Farber/Boston Children’s Cancer and Blood Disorder Center and Harvard Medical School, Boston, Massachusetts, USA.; 4 Department of Urology, Comprehensive Cancer Center, Medical University of Vienna, Vienna, Austria.; 5 Department of Oncologic Pathology and; 6 Center for Patient Derived Models, Dana-Farber Cancer Institute, Harvard Medical School, Boston, Massachusetts, USA.; 7 Department of Radiation Oncology, Tufts Medical Center, Boston, Massachusetts, USA.; 8 Department of Medicine and; 9 Masonic Cancer Center, University of Minnesota-Twin Cities, Minneapolis, Minnesota, USA.

**Keywords:** Oncology, Brain cancer, Cancer, Molecular genetics

## Abstract

Collateral lethality occurs when loss of a gene/protein renders cancer cells dependent on its remaining paralog. Combining genome-scale CRISPR/Cas9 loss-of-function screens with RNA sequencing in over 900 cancer cell lines, we found that cancers of nervous system lineage, including adult and pediatric gliomas and neuroblastomas, required the nuclear kinase vaccinia-related kinase 1 (VRK1) for their survival in vivo. *VRK1* dependency was inversely correlated with expression of its paralog *VRK2*. *VRK2* knockout sensitized cells to *VRK1* loss, and conversely, *VRK2* overexpression increased cell fitness in the setting of *VRK1* loss. DNA methylation of the *VRK2* promoter was associated with low *VRK2* expression in human neuroblastomas and adult and pediatric gliomas. Mechanistically, depletion of *VRK1* reduced barrier-to-autointegration factor phosphorylation during mitosis, resulting in DNA damage and apoptosis. Together, these studies identify VRK1 as a synthetic lethal target in *VRK2* promoter–methylated adult and pediatric gliomas and neuroblastomas.

## Graphical abstract

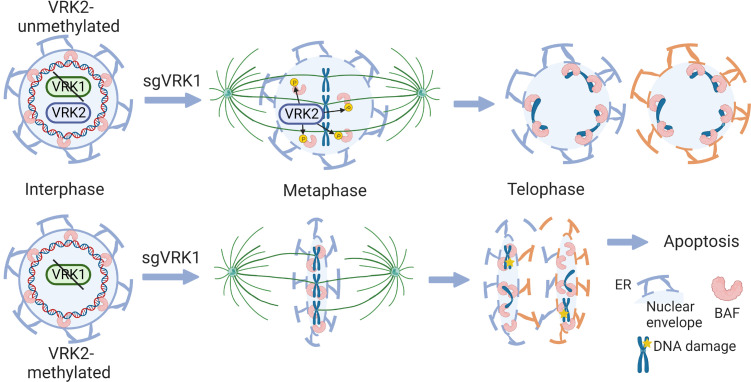

## Introduction

Tumors of the nervous system constitute some of the most devastating malignancies in both adult and pediatric patients (1). Tumors arising in the central and peripheral nervous system (CNS and PNS, respectively) often exhibit an aggressive clinical course and are refractory to currently available systemic therapy (2, 3).

Glioblastoma is the most common primary brain tumor in adults and is characterized by poor prognosis and low cure rates (4). Diffuse midline gliomas (DMGs) that harbor histone 3 lysine27-to-methionine (H3 K27M) mutations occur in children with a peak incidence of 6 to 9 years of age (5). Due to their infiltrative growth pattern, these gliomas are unresectable and uniformly fatal. Neuroblastoma (NB) is the most common extracranial solid tumor malignancy in childhood, commonly originating in the adrenal medulla or paraspinal ganglia (2). Standard-of-care treatment schemes for these tumors remain cytotoxic radiochemotherapy and surgery, and patient prognosis has not substantially improved over the last decade (3, 6, 7). Thus, there is an urgent need for the identification of novel, targetable biomarkers in these tumor entities to translate into improved patient outcomes.

The implementation of CRISPR/Cas9 screening technologies has facilitated systematic studies to identify novel therapeutic targets and biomarkers of response across many cancers. Such dependency maps have unveiled a number of gene targets beyond known oncogenic drivers and hold the potential for tumor-specific, personalized therapy (8). Integration of genome-scale functional studies with genome-wide transcriptomics or epigenomics allows for correlative connections between gene dependencies and cancer transcriptional landscapes.

By comprehensively integrating genome-scale, loss-of-function genetic screens with RNA sequencing and analysis of DNA methylation patterns, we identified the nuclear serine/threonine kinase vaccinia-related kinase 1 (VRK1) as a highly selective dependency in adult and pediatric CNS and PNS tumors that exhibit low expression of the *VRK1* paralog *VRK2*.

## Results

### VRK1 is a selective dependency in adult and pediatric glioma and NB.

**Figure 1:**
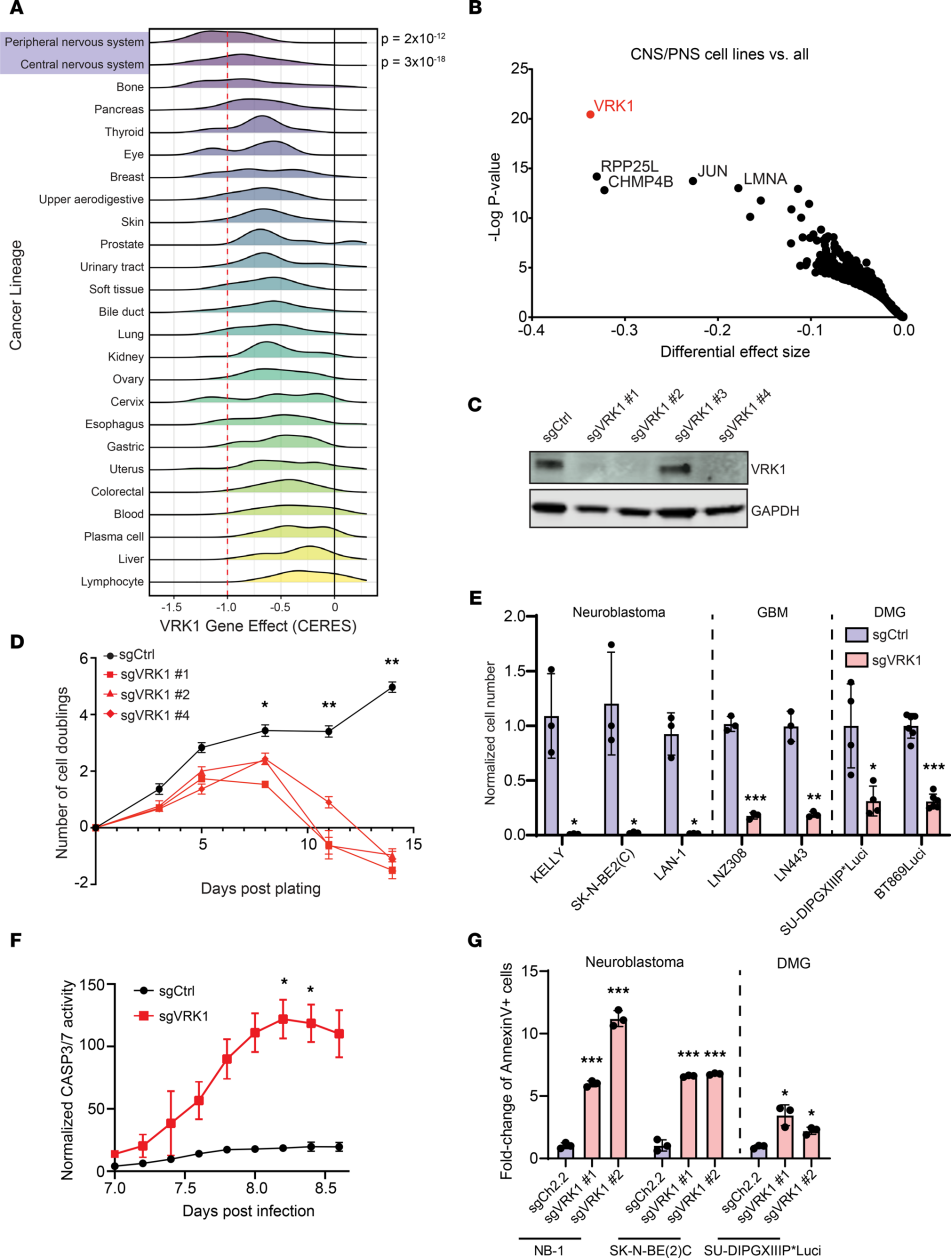
VRK1 is a dependency in glioblastoma, NB, and DMG. (**A**) Histogram plots showing *VRK1* CERES-corrected dependency scores in over 900 cell lines, representing 25 cancer lineages from the DepMap data set (21Q3). Compared with all other lineages, cell lines in the CNS (*P* = 2 × 10^–12^) and PNS (*P* = 3 × 10^–18^) lineages were significantly more dependent on *VRK1*. (**B**) Differential dependency of gene KO in CNS and PNS cell lines versus all other lineages. Gene effect size is calculated as the difference in average CERES score between lineage groupings, and *q* value is determined by limma eBayes methodology. The top enriched dependencies in CNS/PNS lineages are annotated. (**C**) VRK1 protein expression following expression of 4 different sgRNAs in the NB-1 neuroblastoma cell line. The top 3 guides with greatest VRK1 loss were carried forward in subsequent experiments. (**D**) Population doubling assay following *VRK1* KO with 3 separate guides in NB-1 cells. sgCtrl represents a nontargeting control guide (*n* = 3; mean ± SD). (**E**) sg*VRK1* KO after 14 days in cell lines representing NB (*n* = 3), GBM (*n* = 2), and DMG (*n* = 2) models. (*n* ≥ 3; mean ± SD plotted.) (**F**) Time course of CASP3/7 activity, as measured by cleavage of a peptide reporter, following *VRK1* KO in LN443 cells (*n* = 3; mean ± SD). Total reporter fluorescence signal is normalized by cell confluence. Significance at each time point was determined by 2-way ANOVA (treatment × time). *P < 0.05. (**G**) Quantification of annexin V–positive cells following *VRK1* KO with 2 separate guides in 3 cell lines representing NB and DMG lineages after 7 days. (*n* = 3; mean ± SD; from 2 separate experiments.) *P < 0.05, **P < 0.001, ***P < 0.0001; significance was determined by 2-tailed Student’s *t* test (**E**) and 1-way ANOVA with Tukey’s (**D** and **G**).

The Cancer Dependency Map (DepMap) includes CRISPR/Cas9 loss-of-function screens performed in over 900 cell lines representing 25 cancer lineages (9). Using this data set, we found that *VRK1* was a strong genetic dependency in the adult glioma (*P* = 2 × 10^–12^; Student’s *t* test; *n* = 61) and pediatric NB (*P* = 3 × 10^–8^; Student’s *t* test; *n* = 20) lineages (Figure 1A). Indeed, *VRK1* was the gene with the highest differential dependency in CNS and PNS lineages as compared with all other tumors (Figure 1B).

We validated that VRK1 was required for cell proliferation using CRISPR/Cas9 KO. Three of the 4 *VRK1* sgRNAs that were included in the DepMap led to robust VRK1 KO and proliferation defects in NB, DMG, and glioblastoma multiforme (GBM) cultures (Figure 1C and Supplemental Figure 1, A–C; supplemental material available online with this article; https://doi.org/10.1172/jci.insight.158755DS1). *VRK1* KO resulted in a significant decrease in cell fitness approximately 8 days following viral transduction in neuroblastoma NB-1 cells (Figure 1D), as well as in a larger panel of NB and GBM cell models (Figure 1E). In addition, the DepMap includes a pediatric glioma model (KNS42), which also demonstrated strong *VRK1* dependency. We therefore tested primary pediatric histone H3 K27M DMG neurosphere models and verified that *VRK1* single-gene KO significantly decreased cell viability (Figure 1E). To determine whether the reduced viability was due to apoptosis, we performed live-cell experiments with a caspase-3/7 (CASP3/7) cleavage reporter in the LN443 GBM cell line and found significantly higher CASP3/7 activity after *VRK1* KO (Figure 1F and Supplemental Figure 1D). In an orthogonal approach, we found significant induction of apoptosis in NB and DMG models following *VRK1* KO, as assessed by annexin V/propidium iodide (PI) staining (Figure 1G and Supplemental Figure 1E). We failed to observe significantly altered cell cycle profiles in response to *VRK1* KO in GBM or DMG models and observed only a small increase of cells in G2/M phase in NB cells (Supplemental Figure 2, A–D). Taken together, these observations demonstrate that *VRK1* is a robust dependency in tumors of nervous system lineages, and *VRK1* KO results in apoptotic cell death.

### VRK2 expression is a biomarker for VRK1 dependency.

**Figure 2:**
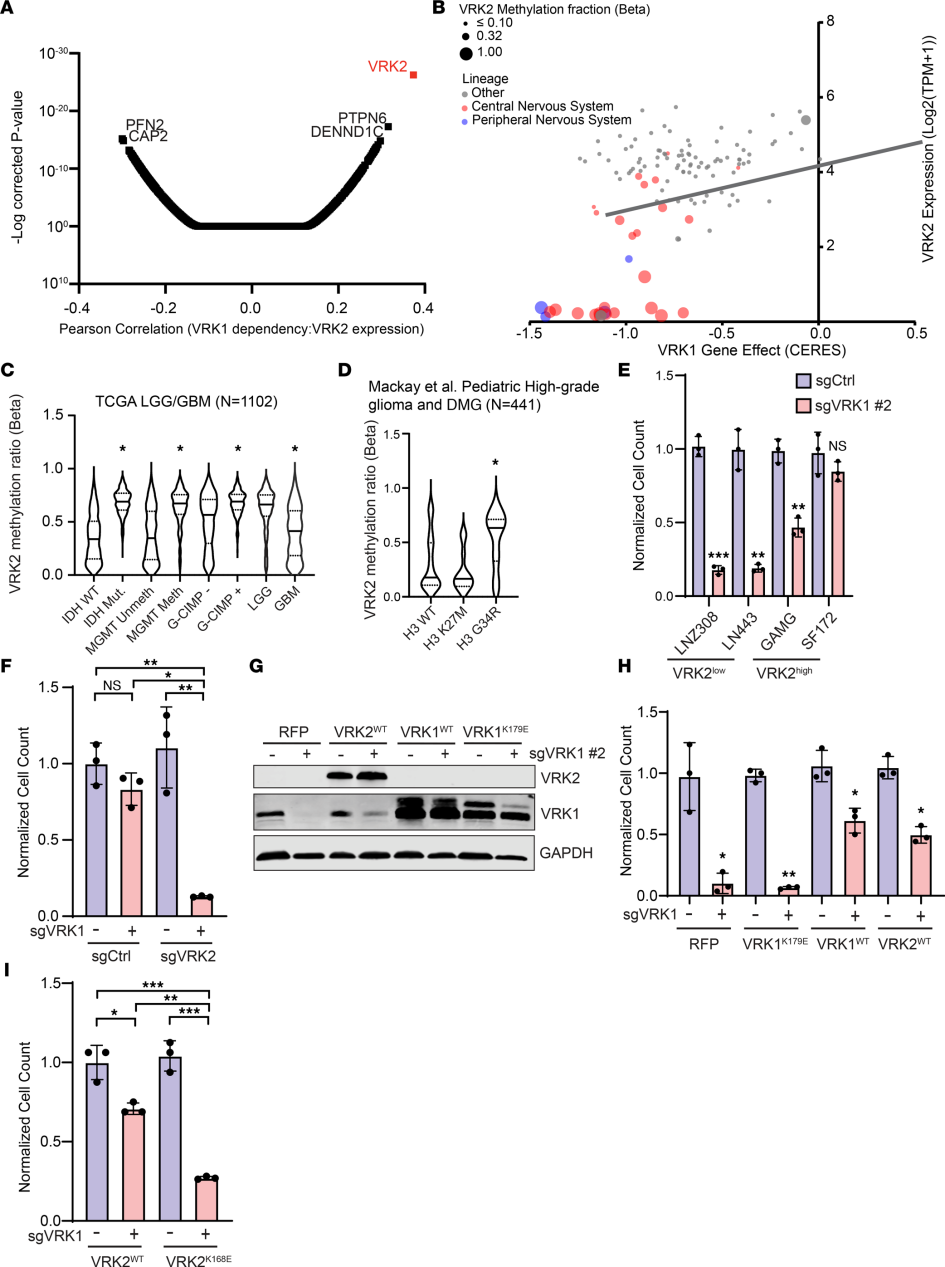
*VRK1* dependency is correlated with *VRK2* expression. (**A**) Whole-genome Pearson correlations between gene expression from CCLE (21Q3) and *VRK1* dependency in the DepMap database (21Q3) and adjusted *P* values. (**B**) Scatterplot showing *VRK1* dependency versus *VRK2* expression. Extent of *VRK2* promoter methylation is indicated by dot size. Red dots represent cell lines of CNS lineage, and blue dots represent PNS lineage. (**C**) *VRK2* promoter methylation status stratified by clinical characteristics across the TCGA GBM-LGG cohort. LGG, low grade glioma; GBM, glioblastoma multiforme. Violin plots with mean (solid line) and first and third quartiles (dashed line). (**D**) *VRK2* promoter methylation in pediatric high-grade gliomas and DMGs with wild-type histone H3 and mutant histone H3 (K27M or G34R). Data from Mackay et al., 2017 (39). Violin plots with mean (solid line) and first and third quartiles (dashed line). (**E**) Cell viability following 14 days’ KO of *VRK1* in *VRK2*^lo^ LNZ308 and LN443 cell lines and *VRK2*^hi^ GAMG and SF172 cell lines. (**F**) Cell viability analysis 14 days following *VRK1* KO in *VRK2*^hi^ GBM cell line (SF172), expressing control CRISPR sgRNA or sgRNA targeting *VRK2*. (*n* = 3; mean ± SD.) (**G**) Immunoblot showing the overexpression of exogenous VRK2^WT^, VRK1^WT^, and kinase-inactive VRK1^K179E^ in NB-1 NB cells with or without *VRK1* KO. RFP, red fluorescent protein. (**H**) Cell viability analysis for NB-1 cells in **G** following 14 days of *VRK1* KO in cells overexpressing VRK2^WT^, VRK1^WT^, and kinase-inactive VRK1^K179E^. (*n* = 3; mean ± SD.) (**I**) Effect of VRK2^WT^ or VRK2^K168E^ overexpression on LN443 GBM cell viability following 14 days *VRK1* KO. (*n* = 3; mean ± SD.) *P < 0.05, **P < 0.001, ***P < 0.0001; significance was determined by 2-tailed Student’s *t* test (**E**) and 1-way ANOVA with Tukey’s test (**C**, **D**, **F**, **H**, and **I**).

To identify genes or pathways that predict *VRK1* dependency, we correlated gene expression data from the Cancer Cell Line Encyclopedia (CCLE) with *VRK1* dependency and found that it was most strongly correlated with the loss of expression of its paralog *VRK2* (Pearson correlation = 0.37, *q* < 10^–25^) (Figure 2A). VRK2 has 2 known functional isoforms, VRK2A and VRK2B. VRK2A contains a C-terminal domain that anchors the protein to the endoplasmic reticulum, while alternative splicing of the VRK2B isoform results in the loss of the C-terminal domain and primarily nuclear localization. We evaluated whether the VRK1 dependency could be explained by isoform-specific expression. We observed that expression of the *VRK2A* and *VRK2B* isoforms strongly correlated with each other (Supplemental Figure 3A) and that *VRK2A* represented the majority of total *VRK2* transcripts in tumor cell lines (Supplemental Figure 3B). The correlation of VRK2 isoform expression with the VRK1 dependency showed that both isoforms predict VRK1 dependency, though the magnitude of difference in *VRK2A* expression more clearly discerns the VRK2^lo^ population (Supplemental Figure 3, C and D).

We next used Celligner, which integrates RNA-sequencing data from The Cancer Genome Atlas (TCGA), Treehouse Childhood Cancer Initiative (Treehouse), and Office of Cancer Genomics Therapeutically Applicable Research to Generate Effective Treatments (TARGET) human tumor-sequencing studies, to identify whether low *VRK2* expression correlates with nervous system lineage cancers (10). We found that CNS and PNS tumors exhibited the lowest expression of *VRK2* across all tumor lineages, while *VRK1* expression was slightly reduced in CNS/PNS tumors as compared with all other tumor lineages (Supplemental Figure 4, A and B). Within NB, we found that *VRK2* was not differentially expressed among high-*MYCN*-expressing tumors and was slightly elevated in NB tumors enriched for a mesenchymal (MES) gene expression program. However, we noted that *VRK2* was still repressed as compared with all other tumor lineages (Supplemental Figure 4C). We also found that in primary DMG models, VRK2 was not expressed significantly (Supplemental Figure 4D). These observations suggest that *VRK2* is expressed at low levels in human cancers of the nervous system, mirroring the CCLE data.

Transcriptional repression is enforced through epigenetic regulation, including methylation of cytosine-phosphate-guanine (CpG) dinucleotides at gene promoters (11). We observed that low levels of *VRK2* RNA expression were associated with *VRK2* promoter CpG methylation as determined by reduced representation bisulfite sequencing microarrays, and *VRK2* was enriched in CNS and PNS lineages (Figure 2B). We confirmed, by bisulfite sequencing, widespread CpG methylation at the *VRK2* promoter in a panel of DMG, GBM, and NB cell lines that exhibited low *VRK2* expression (Supplemental Figure 5A). Cell lines that expressed higher levels of *VRK2* did not exhibit a similar pattern of CpG methylation. In addition, we found a strong association of CpG island probe methylation with *VRK2* expression when we analyzed a cohort of GBM patient tumors (Supplemental Figure 5B). Gene expression data from healthy neural tissue also demonstrated low *VRK2* expression relative to other tissues, suggesting *VRK2* promoter methylation may be specific to the neural lineage (Supplemental Figure 5C).

Because of the link between *VRK2* methylation and expression, we next wanted to evaluate whether *VRK2* methylation can be detected in primary human tumors. Using methylation array data from TCGA low-grade and high-grade gliomas data set, we identified robust *VRK2* promoter methylation across subtypes that occurred more frequently in tumors that exhibited isocitrate dehydrogenase (IDH) mutations, O6-methylguanine-DNA methyltransferase (*MGMT*) methylation, the CpG island methylator phenotype (G-CIMP), or lower grade (Figure 2C). In a separate data set of over 1,000 pediatric high-grade gliomas, including DMGs, we found *VRK2* promoter methylation in subsets of histone 3 wild-type and H3 K27M tumors, but *VRK2* promoter methylation was most highly associated with the histone H3 G34R mutation (Figure 2D). We conclude that *VRK2* promoter methylation is observable among cancer subtypes and is a predictor for *VRK2* expression and thus a VRK1 dependency.

To verify the synthetic lethal relationship between *VRK1* and *VRK2* experimentally, we focused on a panel of 4 GBM cell lines with heterogeneous expression of VRK2 (Supplemental Figure 6, A and B). Consistent with our observations in the DepMap, we found greater *VRK1* dependency in the 2 VRK2^lo^ cell lines (LNZ308, LN443) than in the 2 VRK2^hi^ cell lines (GAMG, SF172) (Figure 2E).

We then directly tested whether modulation of *VRK2* expression altered the response to *VRK1* KO. To create an isogenic experimental model, we deleted *VRK2* in the VRK2^hi^ SF172 GBM cell line and then introduced either a control or *VRK1* sgRNA. We found that *VRK2* KO sensitized SF172 to subsequent *VRK1* KO (Figure 2F and Supplemental Figure 6, B and C). In contrast, ectopic overexpression of wild-type *VRK1*, insensitive to *VRK1* sgRNAs via synonymous mutations, but not kinase-inactive (12) VRK1^K179E^ rescued *VRK1* KO (Figure 2, G and H; and Supplemental Figure 6, D–G). Similarly, VRK2 overexpression in *VRK2*^lo^ GBM lines and primary DMG neurospheres rescued *VRK1* dependency, which required VRK2 kinase activity, as expression of the kinase-inactive VRK2^K168E^ mutant did not rescue *VRK1* KO–induced cell death (Figure 2, G–I; and Supplemental Figure 6, D–H). Overexpression of either *VRK2A* or *VRK2B* isoforms, and not their kinase-inactive forms, rescued *VRK1* knockout, suggesting that either isoform sufficed to substitute for the loss of VRK1 function (Supplemental Figure 6I). In summary, *VRK1*-dependent cell lines require VRK1 kinase activity for survival. Furthermore, VRK2 can act as a surrogate kinase for VRK1, providing a mechanistic explanation for the observed VRK1 dependency in CNS and PNS tumors with low *VRK2* expression levels.

### Global phospho-proteomics link VRK1 loss to DNA damage and nuclear membrane substrates.

**Figure 3:**
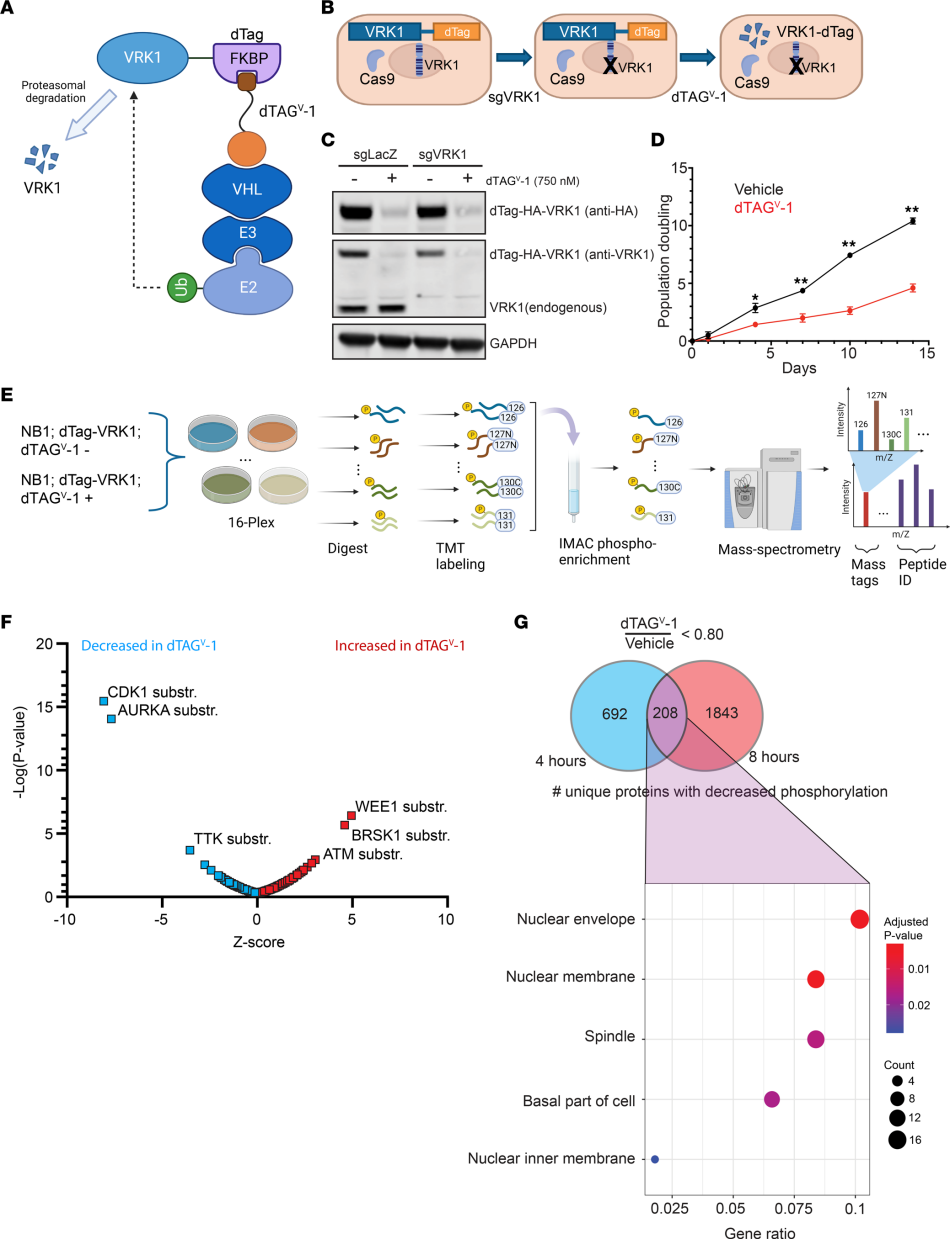
Global phospho-proteomics following acute VRK1 degradation. (**A**) Schematic of dTAG-VRK1 degrader system. The conjugated FKBP12^F36V^ binding domain allows small molecule–mediated (dTAG^V^-1) recruitment of the VHL ubiquitin ligase complex, targeting exogenous VRK1 for proteasomal degradation. (**B**) Schematic of VRK1 degrader experiments. Exogenous dTAG-VRK1 is transduced to rescue CRISPR KO of endogenous *VRK1*. Exogenous dTAG-VRK1 is then under the control of the small molecule degrader (dTAG^V^-1) allowing for acute downregulation. (**C**) Immunoblot validation of the dTAG-VRK1 degrader system in NB-1 neuroblastoma cells. Exogenous dTAG-VRK1 was degraded with dTAG^V^-1. Endogenous *VRK1* was independently targeted with CRISPR KO. sgLacZ is a nontargeting guide control. (**D**) Cell viability analysis of dTAG-VRK1-NB-1 cells following addition of either vehicle control or 0.5 μM dTAG^V^-1. Significance at each time point was determined by 2-way ANOVA (treatment × time). *P < 0.05, **P < 0.001. (**E**) Schematic of the quantitative, global phospho-proteomic experiment. Samples were generated in triplicate at 4 hours and 8 hours after dTAG^V^-1 (0.5 μM) addition. Following trypsin digestion, peptides were tagged with isobaric tandem mass tags (TMTs), then combined. Phospho-enrichment was performed using IMACs, and then peptides were run on an Orbitrap mass spectrometer. MS2 spectra offer peptide IDs and sample deconvolution through attached mass tags. (**F**) KSEA of phosphorylation site dynamics following acute degradation of exogenous VRK1. Kinase substrates of CDK1 and AURKA were significantly downregulated following degradation (blue), while substrates of WEE1, BRSK1, and ATM were significantly upregulated (red). (**G**) Top panel: Venn diagram showing number of unique proteins with a decrease in phosphorylation for at least 1 phosphorylation site in dTAG^V^-1–treated samples. Bottom panel: Dot plots showing the overlap of downregulated protein phosphorylation (208 proteins) with select categories of the C5 MSigDB library. All gene sets have FDR ≤ 0.05 as determined by 1-tailed Fisher’s exact test.

Given the requirement of VRK1 in CNS/PNS tumors, we sought to understand the immediate effects of VRK1 loss. To answer these questions, we designed a degradable VRK1 construct using the dTAG system (13), providing the ability to rapidly deplete exogenous dTAG-VRK1 from cells. Cells were transduced with *VRK1* fused with a C-terminal FKBP12^F36V^ domain (dTAG-VRK1), which can be rapidly degraded with a small molecule (dTAG^V^-1) in a manner dependent on Von Hippel–Lindau (VHL) (Figure 3A). Exogenous expression of dTAG-VRK1 rescued growth defects in the CRISPR KO of endogenous *VRK1* in LN443 (GBM), NB-1 (NB), and Kelly (NB) cells, signifying that the fusion protein itself had no effect on canonical VRK1 function (Figure 3, B and C, and Supplemental Figure 7A). However, addition of dTAG^V^-1 and subsequent degradation of dTAG-VRK1 resulted in significantly reduced cell viability in *VRK1*-dependent cell lines, establishing a functional system to rigorously examine mechanisms underlying VRK1 dependency (Figure 3D and Supplemental Figure 7B).

To identify downstream effectors/pathways of VRK1 kinase, we performed quantitative phospho-proteomics using the dTAG-VRK1 degrader system (Figure 3E). dTAG-VRK1-NB-1 cells were treated with dTAG^V^-1 to identify early phosphorylation changes following degradation of VRK1. Following cell lysis, isobaric tandem mass tags (TMTs) allowed for deconvolution of pooled samples and relative quantitation among the samples. Phosphorylated peptides were enriched using immobilized metal affinity columns (IMACs) and analyzed by mass spectrometry. We performed kinase-substrate enrichment analysis (KSEA) of phospho-peptide dynamics following either 4 or 8 hours of acute dTAG-VRK1 degradation (14). We found that substrates of cell cycle and mitotic kinases (CDK1 and AURKA) were downregulated, while substrates of DNA damage response kinases (ATM and WEE1) were upregulated (Figure 3F). A total of 208 phospho-proteins were downregulated at both the 4-hour and 8-hour time points. Gene set enrichment analysis of these overlapping proteins revealed an enrichment of proteins associated with the nuclear envelope and spindle assembly (Figure 3G). Notably, members of the inner nuclear membrane LEM domain family of proteins, including LEMD3, EMD, and TMPO, showed at least 1 phosphorylation site that was significantly reduced upon VRK1 degradation (Supplemental Figure 8). Overall, these observations suggest a critical role of VRK1-regulated pathways in mitosis, nuclear envelope and chromatin homeostasis, as well as DNA damage, in CNS and PNS cell models.

### VRK1 and VRK2 loss leads to postmitotic nuclear membrane deficits and DNA damage.

**Figure 4:**
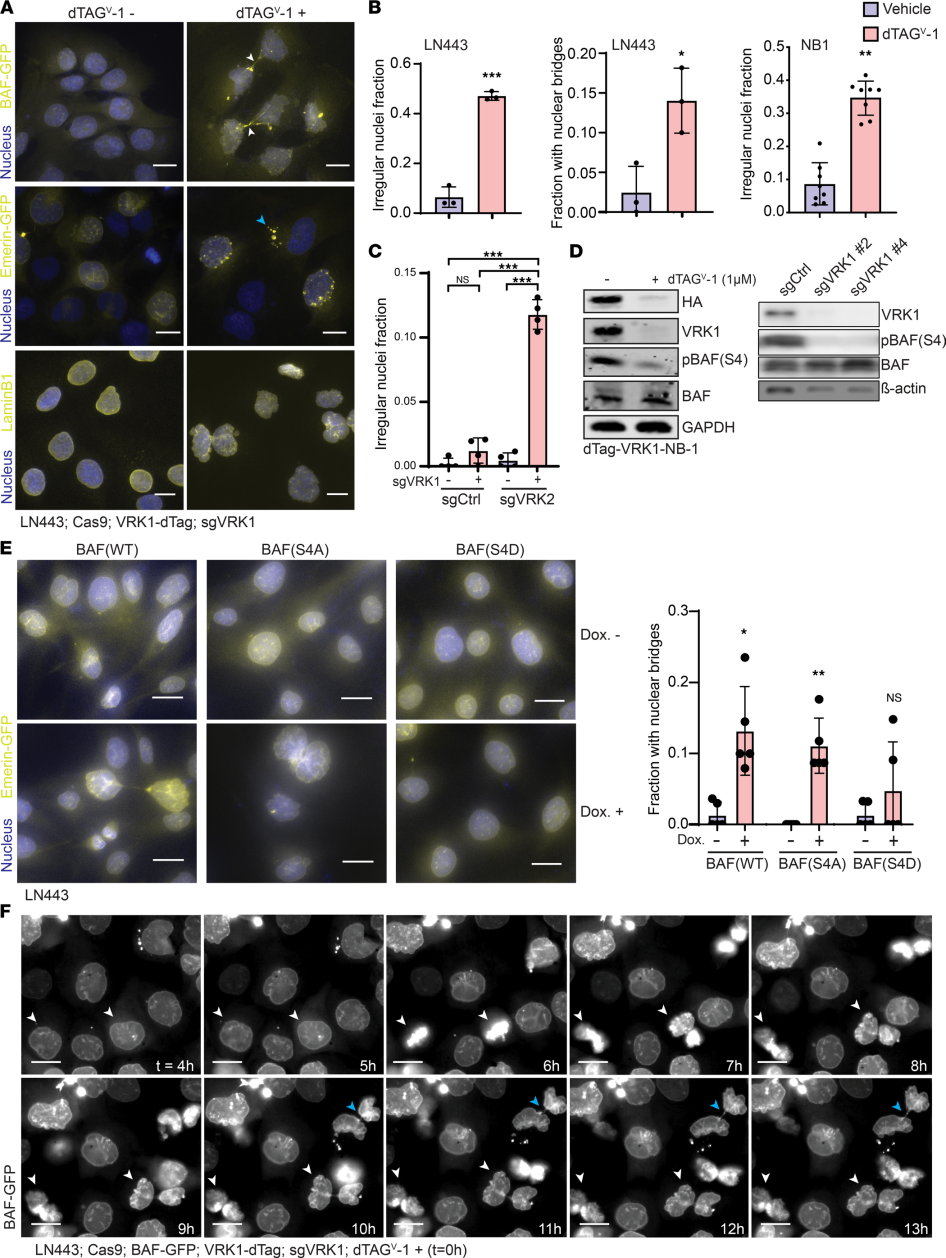
VRK1 loss is associated with nuclear envelope malformation. (**A**) Nuclear membrane morphology in the LN443 GBM cell line following exogenous VRK1 degradation by dTAG^V^-1 after 1 day. White arrows point to nuclear bridges. Blue arrow points to micro-nuclei. (**B**) Left: Quantitation of irregular nuclei, by LaminB1 staining, following VRK1 degradation as seen in **A** (*n* = 3 fields of >50 cells each; mean ± SD). Center: Quantitation of nuclear bridges following VRK1 degradation as seen in **A** (*n* = 3 fields of > 50 cells each; mean ± SD). Right: quantitation of irregular nuclei following VRK1 degradation in the NB-1 neuroblastoma cell line expressing GFP-BAF seen in Supplemental Figure 9D (*n* = 8 fields of >50 cells each; mean ± SD). (**C**) Quantitation of irregular nuclei, by LaminB1 staining, following KO of both *VRK1* and *VRK2* in SF172 as seen in Supplemental Figure 9A. (*n* = 4 fields of >50 cells each; mean ± SD.) (**D**) Immunoblot of phosphorylated BAF (S4) and total BAF following dTAG^V^-1 treatment in dTAG-VRK1-NB-1 cells (left panel) or KO of *VRK1* with 2 independent sgRNAs in BT869Luci DMG neurospheres (right panel). Represents 2 independent experiments. (**E**) Left panel: Nuclear envelope morphology (Emerin-GFP) following doxycycline-induced expression of BAF mutants in LN443 GBM cell line after 3 days: wild-type (WT), S4A (nonphosphorylatable), S4D (phospho-mimetic). Right panel: Quantitation of nuclear bridging phenotype in LN443 cell lines expressing BAF mutants (*n* = 3; mean ± SD). (**F**) Live-cell, time-lapse experiment showing nuclear envelope morphology following VRK1 degradation in LN443 (dTAG^V^-1 addition at *t* = 0 hours). White arrows point to cells undergoing mitosis. Blue arrows point to chromatin bridges. Represents 2 independent experiments. Scale bars: 20 μm. *P < 0.05, **P < 0.001, ***P < 0.0001; significance was determined by 2-tailed Student’s *t* test (**B**) and 1-way ANOVA with Tukey’s test (**C** and **E**).

Phospho-proteomic analysis following VRK1 degradation strongly suggested that VRK1 loss alters the phosphorylation of protein substrates in the nuclear membrane. To visualize nuclear membrane dynamics following VRK1 degradation, we transduced cells with GFP-labeled nuclear lamina-associated proteins: barrier-to-autointegration factor (BAF) and emerin. In addition, we stained cells with anti-LaminB1/2 antibody. Twenty-four hours following degradation of dTAG-VRK1, the nuclear membrane of LN443 GBM cells became misshapen with the formation of lobes and ruffling as well as chromatin bridging between nuclei (Figure 4, A and B). In concordance with reduced cell viability, KO of both *VRK1* and *VRK2* in *VRK2*^hi^ cells (SF172) increased irregular nuclei compared with individual kinase KO alone (Figure 4C and Supplemental Figure 9A).

The nuclear envelope protein BAF serves to tether chromatin to proteins in the inner nuclear membrane and is a substrate of both VRK1 and VRK2 individually on the serine-4 (S4) residue (15, 16). During mitosis, phosphorylation of BAF on S4, and subsequent nuclear lamina-DNA untethering, are required for mitotic chromosome segregation, as well as postmitotic nuclear envelope reassembly (16). BAF(S4) phosphorylation was not detectable in our phospho-proteomic analysis; however, the observed reduced phosphorylation of LEM domain proteins that bind to BAF (Supplemental Figure 8) led us to hypothesize that the altered nuclear envelope dynamics observed upon *VRK1* KO was due to decreased phosphorylation of BAF. Indeed, dTAG^V^-1–mediated degradation of exogenous VRK1 in dTAG-VRK1-NB-1 cells and CRISPR KO of *VRK1* in the *VRK2*^lo^ DMG neurosphere models BT869Luci and SU-DIPGXIIIP*Luci strongly decreased levels of phosphorylated BAF(S4) but not total BAF (Figure 4D and Supplemental Figure 9B). We tested whether ectopic overexpression of the nonphosphorylatable mutant BAF^S4A^ would mimic *VRK1/2* loss, and indeed, following doxycycline-induced expression, we observed similar nuclear bridges and distorted nuclear envelope morphology (Figure 4E and Supplemental Figure 9C). We also found that doxycycline-induced ectopic overexpression of BAF^WT^ resulted in the same phenotype, perhaps by saturating the phosphorylation capacity of VRK1/2, leading to a shift in the pool of BAF toward its unphosphorylated form (Figure 4E and Supplemental Figure 9C). In contrast, overexpression of the phospho-mimetic mutant BAF^S4D^ had no effect on nuclear morphology (Figure 4E and Supplemental Figure 9C), suggesting that the S4 phosphorylation site plays a crucial role in VRK1 kinase dependency. Using live-cell imaging, we followed BAF dynamics after dTAG-VRK1 degradation in LN443 GBM cells (Figure 4F and Supplemental Video 1). We observed the same nuclear envelope ruffling and bridging in cells immediately following mitosis. We also found similar phenotypes in NB-1 neuroblastoma cells, where nuclear membrane ruffling predominated (Supplemental Figure 9D). Taken together, these findings indicate that BAF(S4) phosphorylation by VRK1 is essential for CNS and PNS tumor cells to maintain the integrity of nuclear envelope structure and function.

**Figure 5:**
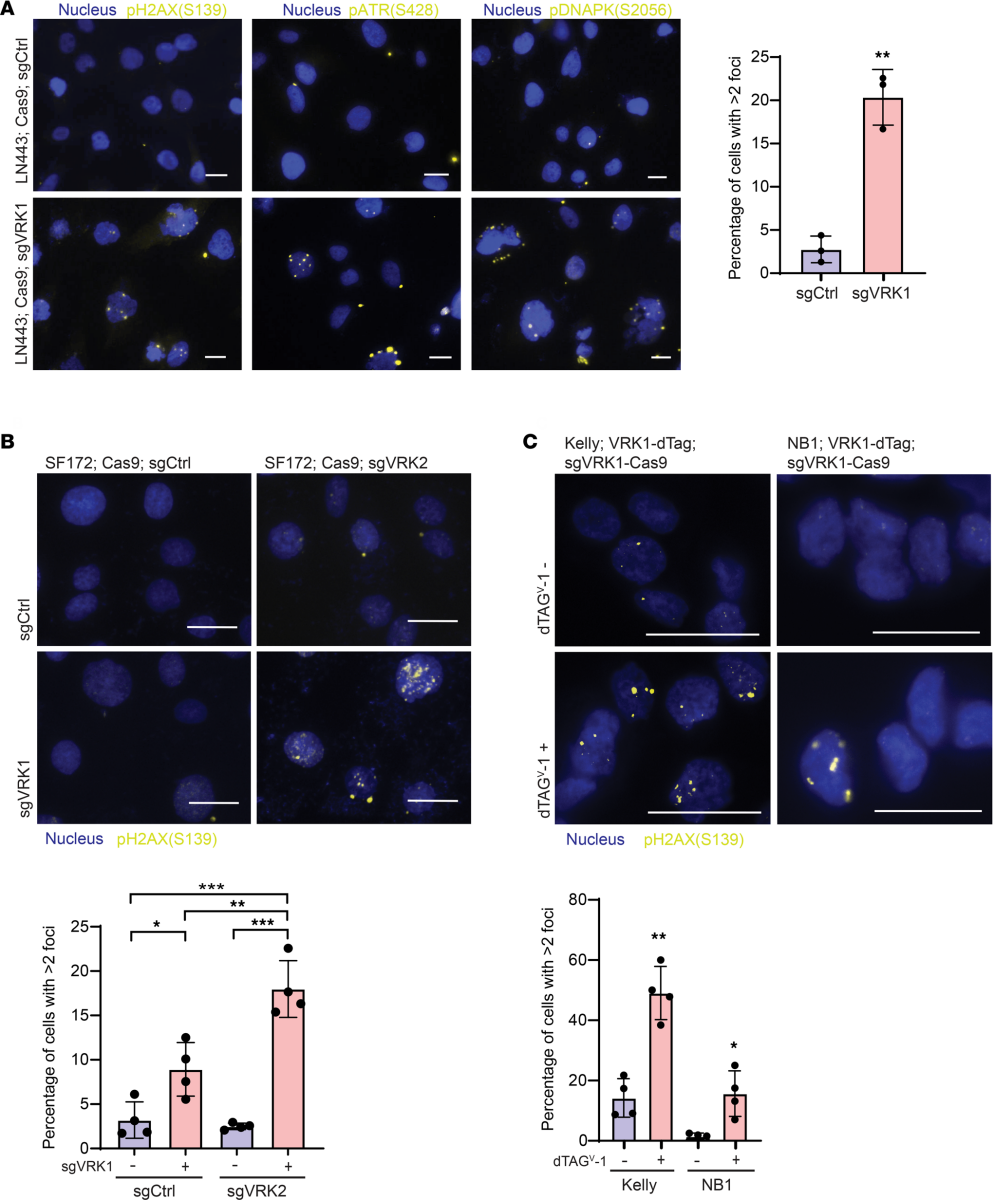
VRK1 loss results in DNA damage. (**A**) Left panel: Nuclear foci of a panel of DNA damage markers — phospho-H2AX (S139), phospho-ATR (S428), and phospho-DNAPK (S2056) — following KO of *VRK1* in LN443 GBM cells for 7 days. Right panel: Quantitation of percentage of cells with >2 phospho-H2AX foci following *VRK1* KO (*n* = 3 fields of >50 cells each; mean ± SD). (**B**) Top panel: Phospho-H2AX foci following 7-day double-KO combinations of sgCtrl/sgCtrl, sgCtrl/sgVRK1, sgCtrl/sgVRK2, and sgVRK1/sgVRK2. Bottom panel: Quantitation of percentage of cells with >2 phospho-H2AX foci following these double-KO combinations (*n* = 4 fields of >50 cells each; mean ± SD). (**C**) Top panel: Phospho-H2AX foci following VRK1 degradation with 0.5 μM dTAG^V^-1 in both Kelly and NB-1 NB cell lines. Bottom panel: Quantitation of percentage of cells with >2 phospho-H2AX foci following dTAG^V^-1 addition (*n* = 4 fields of >30 cells each; mean ± SD). Scale bars: 20 μm. *P < 0.05, **P < 0.001, ***P < 0.0001; significance was determined by 2-tailed Student’s *t* test (**A** and **C**) and 1-way ANOVA with Tukey’s test (**B**).

In addition to observing altered protein phosphorylation at a number of proteins in the nuclear envelope, we also noted an enrichment for substrates of the DNA damage pathways (i.e., substrates of ATM and WEE1) (Figure 3F). Therefore, we performed imaging of DNA damage response foci. At 7 days following KO of *VRK1*, we found an increased number of phospho-histone H2AX foci (S139), phospho-ATR (S428), and phospho-DNAPK (S2056), representing induction of both nonhomologous end-joining and homologous recombination pathways of DNA double-strand break repair (Figure 5A). Corroborating potentiated apoptosis induction, concomitant KO of *VRK1* and *VRK2* increased DNA damage foci (phospho-histone H2AX) in *VRK2*^hi^ GBM cells (Figure 5B) and in 2 NB cell lines after degradation of dTAG-VRK1 (Figure 5C).

### VRK1 is a dependency in tumor models in vivo.

**Figure 6:**
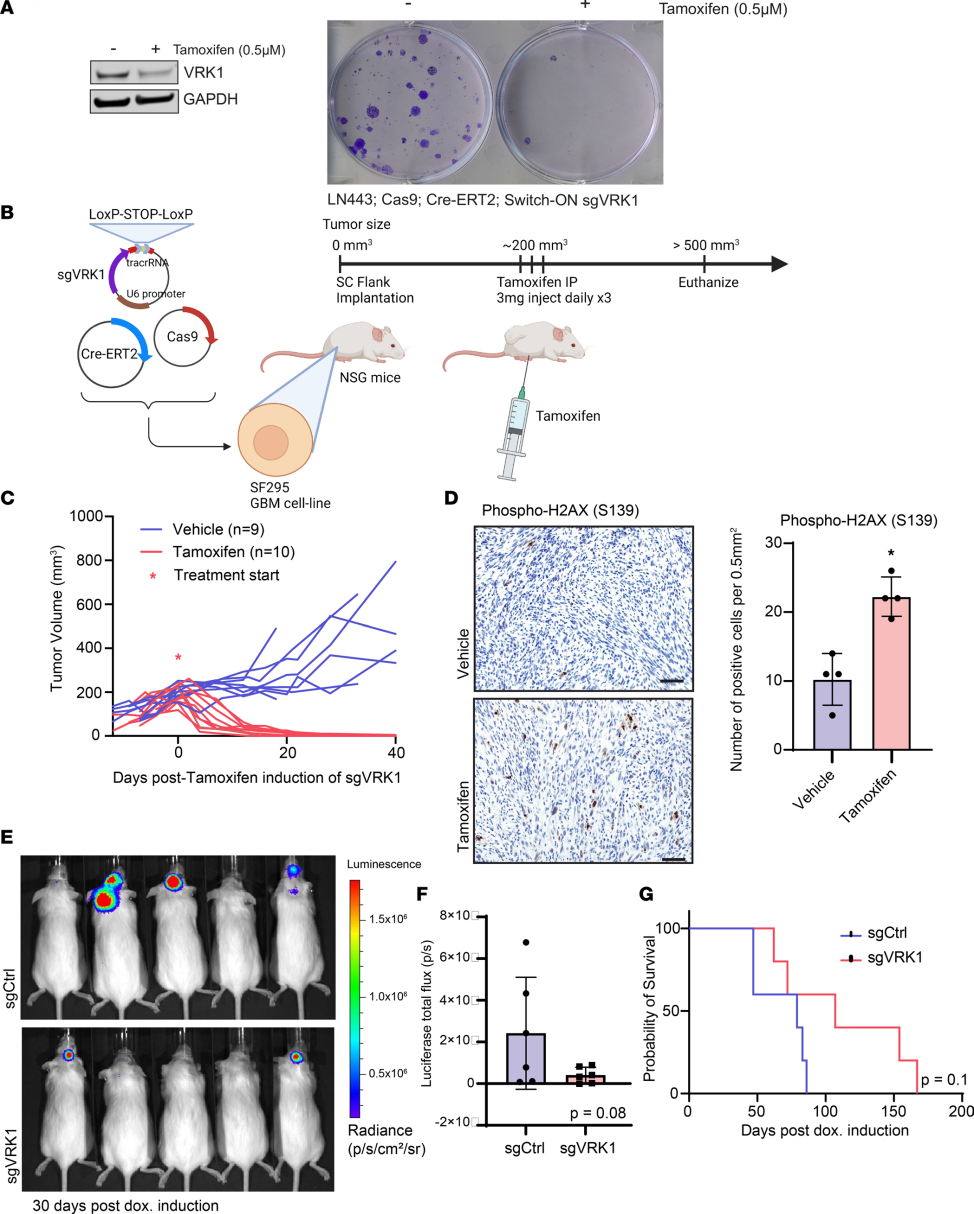
VRK1 is a dependency in vivo. (**A**) Left panel: Immunoblot of VRK1 following tamoxifen-induced expression of sgVRK1 in LN443 cells. Right panel: Clonogenic assay in LN443 cells 14 days following tamoxifen-induced KO of *VRK1*. (**B**) Schematic of the in vivo xenograft experiment. The SF295 GBM cell line was transduced with Cas9, Cre-ERT2, and Switch-ON guide plasmids and implanted in NSG mouse flanks. When the tumors reached a prespecified size (200 mm^3^), the mice were treated with tamoxifen. When the tumor size reached approximately 500 mm^3^ or 40 days following treatment, the mice were euthanized. (**C**) Tumor volume measurements over time of the flank xenografts. * represents injection of tamoxifen or corn oil vehicle control. (**D**) Left panel: representative H&E sections of tumors taken from xenografted mice, 7 days following treatment with tamoxifen or vehicle control (scale bar: 50 μm). Sections were stained with an antibody against phospho-H2AX. Right panel: quantitation of number of phospho-H2AX–positive cells per 0.5 mm^2^ in flank xenografts following tamoxifen or vehicle treatment (*n* = 4 fields; mean ± SD) (*P < 0.05; 2-tailed Student’s *t* test). (**E**) Representative bioluminescence imaging of intracranial xenografts of primary DMG neurospheres with doxycycline-inducible control versus *VRK1* targeting guides taken 30 days after doxycycline induction. (**F**) Quantification of bioluminescence images from **E** (sgCtrl vs. sgVRK1, *P* = 0.08). (**G**) Kaplan-Meier survival curves showing overall survival for mice injected with sgCtrl or sgVRK1 DMG neurospheres into the cranium. Significance was determined by log-rank test (sgCtrl vs. sgVRK1, *P* = 0.10).

To evaluate VRK1 dependency in vivo, we used a tamoxifen-inducible CRISPR/Cas9 system (17). Plasmids expressing Cas9, Cre-ERT2, and the pLenti_Switch-ON guide plasmid targeting *VRK1* were transduced into LN443 or SF295 GBM cell lines. The Switch-ON plasmid has CRISPR guide expression suppressed with a *LoxP*-STOP-*LoxP* site. Upon tamoxifen treatment, Cre recombinase is induced, which removes the transcriptional stop and allows expression of the guide RNA. We first validated *VRK1* KO efficiency in vitro and observed decreased viability after VRK1 depletion (Figure 6A). This cell line (SF295; Cas9; Cre-ERT2; pLenti_Switch-ON_sgVRK1) was subsequently injected into flanks of NSG mice (Figure 6B). Once the xenografts reached approximately 200 mm^3^, *VRK1* KO in tumor cells was induced by intraperitoneal administration of tamoxifen. *VRK1* KO resulted in virtually complete and durable tumor remission in all mice (*n* = 10 tumors) 10–20 days following tamoxifen treatment, whereas tumors in vehicle-treated controls continued exponential growth (Figure 6C). We harvested a subset of tumors 7 days following treatment with tamoxifen or vehicle control, stained for phospho-H2AX (S139), and found evidence of increased DNA damage in tumors in which we induced *VRK1* depletion by tamoxifen treatment (Figure 6D). To evaluate the VRK1 dependency in vivo for NB, we introduced doxycycline-inducible, *VRK1*-specific sgRNAs (Supplemental Figure 10A) into the Kelly NB cell line and verified a robust antiproliferative effect in vitro (Supplemental Figure 10B). Tumor cells were subsequently injected into the rear flank of mice randomized into groups receiving vehicle or doxycycline. We found that *VRK1* KO repressed tumor growth (Supplemental Figure 10, C and D).

To extend these findings to patient-derived models, we generated intracranial xenografts of patient-derived DMG neurospheres that express ZsGreen-Luciferase, Cas9, and a doxycycline-inducible guide vector targeting control or *VRK1*. Cells were stereotactically injected into the striatum of NSG mice. We induced *VRK1* deletion by treating these animals with doxycycline. At 30 days postinjection, we observed decreased luciferase signal in sgVRK1 mice as compared with sgCtrl (*P* = 0.08) (Figure 6, E and F). Decreased tumor growth corresponded to increased survival of mice with *VRK1*-KO neurospheres (*P* = 0.1) (Figure 6G). Taken together, we observed in 3 independent models and cancer lineages that *VRK1* depletion leads to tumor repression in vivo, suggesting that VRK1 is a potential therapeutic target in *VRK2* promoter–methylated adult and pediatric gliomas and neuroblastomas.

## Discussion

Synthetic lethal interactions are a potential source of new biomarker-linked targeted cancer therapy. Specifically, synthetic lethal interactions may involve tumor-specific downregulation of a gene or pathway, resulting in sensitivity to inhibition of another gene or pathway. The success of PARP inhibitors in multiple cancers with homologous recombination pathway deficiency provides evidence that this approach can lead to clinical benefit (18, 19). Specifically, *BRCA1/2* mutations in breast cancer result in dependency on the nonhomologous end-joining DNA repair pathway that is exploited by poly(ADP-ribose) polymerase inhibitors such as olaparib (20).

Gene paralogs are potentially promising sources of synthetic lethal interactions as they usually exhibit strong sequence homology and functional redundancy. For example, *ENO1*-deleted GBMs are sensitive to KO of paralog *ENO2*, blocking glycolysis (21). Loss-of-function *ARID1A*-mutant cancers are sensitive to *ARID1B* KO, causing destabilization of the SWI/SNF chromatin remodeling complex (22). Synthetic lethality in the context of paralogs can occur by epigenetic mechanisms as well. For example, in *NXT2*-methylated NB cell lines, NXT1 is required to facilitate stability of the essential RNA-exporting protein NXF1 (23). Targeting paralogs holds the promise of an increased therapeutic ratio as one interaction partner may be a silenced tumor suppressor or may be co-silenced with other tumor suppressors but not affected in normal tissues. Here, we discovered that tumors with low VRK2 expression are dependent on its paralog VRK1. IDH-mutant gliomas, with their hypermethylated phenotype, also exhibit high *VRK2* gene methylation. In fact, *VRK2* promoter methylation is highly enriched in tumors of the CNS and PNS lineages. During development, differential gene methylation is involved in neuronal cell fate determination, neuronal plasticity, and memory formation (24). Such lineage-specific, differential methylation may lead to other synthetic lethal vulnerabilities in cancer.

The VRK family of atypical serine/threonine kinases was initially discovered for homology with vaccinia virus B1 kinase, which is required for viral replication (25). The family branches early from the kinase evolutionary tree and consists of the functional kinases VRK1 and VRK2 and a pseudokinase, VRK3 (12). Clinically, *VRK1* expression has been associated with high grade and poor prognosis in patients with glioma (26), whereas *VRK2* expression is correlated with improved survival in high-grade astrocytoma (27). In the physiological context, VRK1 localizes to the nucleus, where it is thought to phosphorylate substrates involved in DNA damage response (e.g., histone H2AX) and mitosis (e.g., BAF) (12). Previous work showed that VRK1 is the primary kinase that phosphorylates BAF during mitosis (16). BAF phosphorylation removes its association with chromatin and LEM domain–containing proteins of the nuclear envelope, such as emerin. The paralog, VRK2, exists as 2 main isoforms: VRK2A and VRK2B. VRK2B, which is expressed at lower levels, does not have the C-terminal membrane anchor and so is expressed both in the cytoplasm and in the nucleus (28). It has been shown to share substrates with VRK1, namely p53 (28). VRK2A has also been shown to phosphorylate BAF, similar to VRK1, and modulates the association of BAF with the nuclear membrane in mitosis (15). Unlike VRK1, which localizes to the nucleoplasm, VRK2A associates with A-type Lamins of the nuclear envelope. Birendra et al. hypothesized that VRK1 may modulate BAF phosphorylation in the nucleoplasm, while VRK2A modulates BAF at the nuclear envelope (15). This difference in localization may explain the only partial rescue of VRK1 loss by VRK2A that we observed (Figure 2, H and I). Further, while our data showed a robust connection between VRK2 expression and VRK1 dependency, some tumor lineages, like Ewing sarcoma, demonstrated high VRK2 expression and a strong dependency on VRK1, suggesting that VRK1 may also be required for other tumor cell functions in particular contexts. Future work is needed to understand the VRK1 dependency in VRK2^hi^ models.

**Figure 7:**
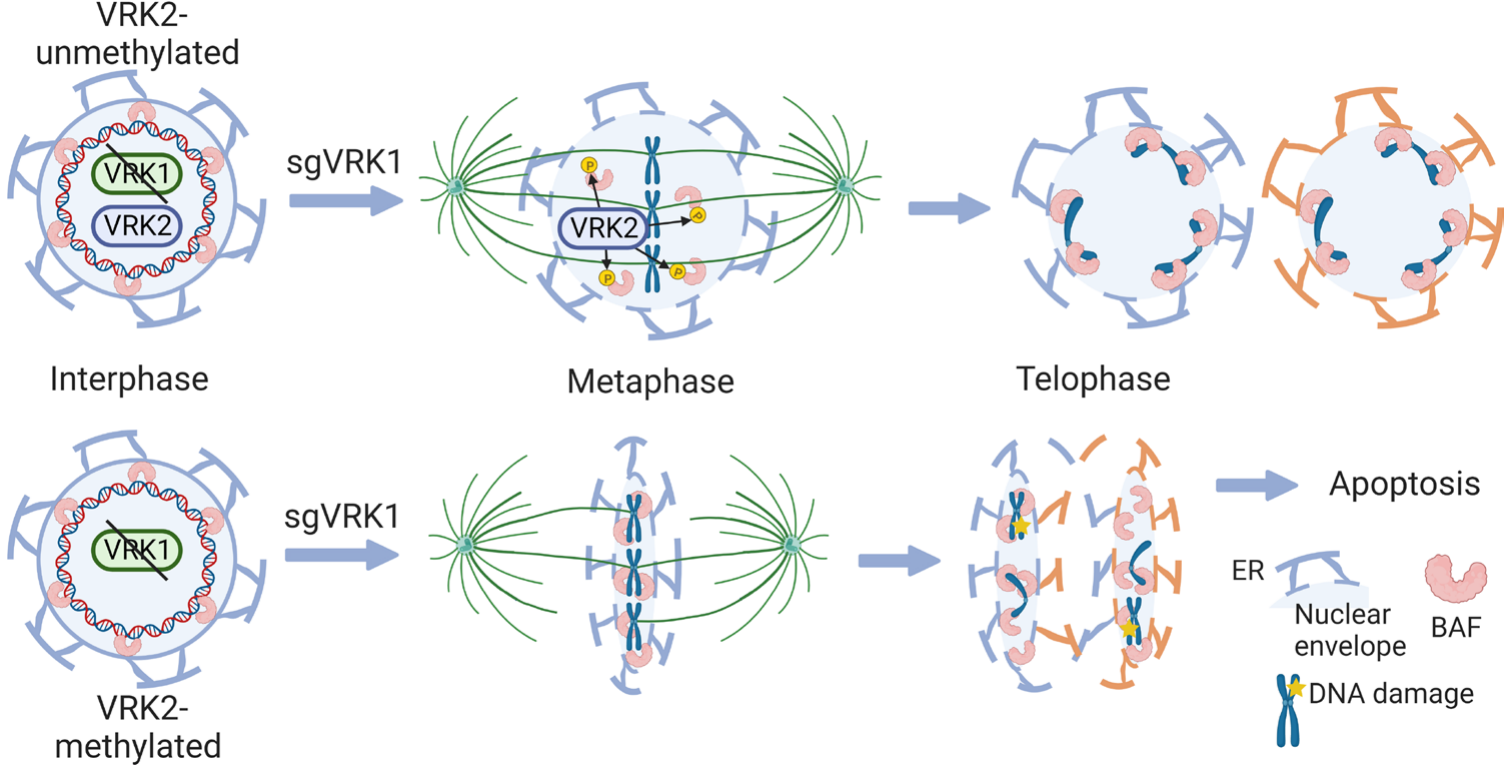
Model for mechanism of synthetic lethality between VRK1 and VRK2. Schematic showing proposed mechanism of synthetic lethality between *VRK1* and *VRK2*. In *VRK2*-unmethylated tumors (top), VRK2 compensates for *VRK1* loss in the phosphorylation of BAF during mitosis. In *VRK2*^lo^ tumors (bottom), loss of *VRK1* leads to retention of BAF during mitosis and the continued association of the nuclear envelope with chromatin. This leads to impaired chromosomal segregation and DNA damage, including nuclear bridging.

Together, our observations are consistent with a synthetic lethal interaction of *VRK1* and *VRK2* (Figure 7). In *VRK2*^hi^ tumors where the *VRK2* promoter is unmethylated, both VRK1 and VRK2 may phosphorylate BAF during mitosis to mediate nuclear envelope disassembly. However, in *VRK2*^lo^ tumors, loss of *VRK1* prevents BAF phosphorylation during mitosis. Thus, our data suggest that VRK1 depletion results in retained association of nuclear envelope fragments with mitotic chromosomes, leading to aberrant nuclear envelope reassembly, nuclear bridging between daughter cells, and ultimately DNA damage and apoptotic cell death. VRK2^lo^ tumors may also be sensitized to DNA-damaging effects, independently of VRK1 loss of function (29). Further research is warranted to investigate the potential for VRK1 inhibitor and DNA-damaging agent combinations in VRK2^lo^ tumors.

Small molecule kinase inhibitors have been investigated for their potential differential effect on VRK1 versus VRK2 activity (30, 31). Vázquez-Cedeira et al. noted that, based on amino acid sequence and protein structural differences from other kinases, both VRK1 and VRK2 are predicted to have low promiscuity and be relatively insensitive to extant kinase inhibitors (30). They further showed that in a small molecule library screen of 20 kinase inhibitors, few molecules decreased VRK1 or VRK2 kinase activity even at high concentrations (100 μM). The compounds that did inhibit kinase activity did so with ATP concentrations 3 orders of magnitude lower than intracellular levels, which the authors noted may limit in vivo use. Recently, a small molecule, based on an aminopyridine scaffold, was developed that showed potent activity against VRK1 in vitro (IC_50_ = 150 nM) (31). However, this compound did not significantly decrease viability in cell culture (31). Potent kinase inhibitors that show differential effect against VRK1 versus VRK2 do not yet exist. A degrader strategy, as modeled in this current study, may represent an alternative approach to targeting VRK1 as a growing number of small molecule degraders (e.g., PROTACs, molecular glues, etc.) targeting specific proteins are undergoing clinical trials in diverse cancers (32).

For VRK1 inhibition to be a viable therapy option, a significant therapeutic ratio is required where normal tissues are spared while cancer cells are targeted. The existence of human genetic variants and mouse transgenic models allows for an approximation of potential on-target toxicities. A rare germline mutation in *VRK1* (R358X) results in lack of VRK1 protein production and manifests in pediatric patients as spinal muscular atrophy with pontocerebellar hypoplasia (33). Although *VRK2* is expressed in most tissues, it has low expression in normal brain tissue, especially the cerebellum, which may explain the CNS phenotype of mutant VRK1. Partial KO of *Vrk1* by gene trapping resulted in a slight reduction in brain size, mild motor dysfunction, and male infertility in mice (34, 35). These findings suggest that side effects of VRK1 inhibition may be tolerated in adults.

In summary, by integrating genome-wide, loss-of-function genetic screens with RNA sequencing and DNA methylation, we identified VRK1 as a selective vulnerability in CNS and PNS cancers with low *VRK2* expression. Taken together, these studies suggest that targeting VRK1 in cancers that harbor *VRK2* promoter–methylated is a potential therapeutic strategy.

## Methods

### Cell culture.

Neuroblastoma (NB-1, Kelly) and GBM (LN443, SF172, GAMG, LNZ308) cell lines were collected from the CCLE and DepMap projects and obtained from the Broad Institute. The cell lines that express pLX_311-Cas9 were generated by Project Achilles (https://depmap.org/portal/achilles) (20). SK-N-BE(2)C were purchased from ATCC. LAN-1 was gifted by Rani George at Dana-Farber Cancer Institute. SK-N-BE(2)C, LAN-1, and GBM cell lines were grown in 10% DMEM (Gibco, Thermo Fisher Scientific) supplemented with glutamine, penicillin, and streptomycin and incubated at 37°C in 5% CO_2_. Kelly and NB-1 were grown in RPMI-1640 (Gibco, Thermo Fisher Scientific) supplemented with 10% FBS and glutamine, penicillin, and streptomycin and incubated at 37°C in 5% CO_2_. Cell lines’ identities were validated by short tandem repeat (STR) profiling, and cells tested negative for mycoplasma with MycoAlert Mycoplasma Detection Kit (Lonza, catalog LT07-418) prior to experimental use. Cell lines used in this study are summarized in Supplemental Table 1.

### Neurosphere culture.

Patient-derived H3 K27M and H3WT glioma neurosphere lines were established at Dana-Farber Cancer Institute (BT869/BT869Luci; available from the Dana-Farber Cancer Institute Center for Patient Derived Models) and Hospital Sant Joan de Deu Barcelona (HSJD-DIPG007, HSJD-GBM001) as previously described (36–38). Neurosphere lines SU-DIPGXIIILuci, SU-DIPGXIIP*Luci, SU-DIPGXXV, SU-pcGBM2, and SU-DIPG48 were a gift from Michelle Monje at Stanford University, Stanford, California, USA. H3 K27M glioma cells were grown as neurospheres in tumor stem media base (38) supplemented with B27 minus vitamin A (Thermo Fisher Scientific), human growth factors (EGF, FGF, PDGF-AA, PDGF-BB from Shenandoah Biotechnology), and heparin (Stemcell Technologies) in ultra-low-attachment flasks. Indicated cell models expressing luciferase were generated as previously described (39). Neurosphere cultures were dissociated for passaging using Accutase cell detachment solution (Stemcell Technologies) for 3–5 minutes at 37°C. All neurosphere models were authenticated by high-resolution STR profiling (Molecular Diagnostics Core, Dana-Farber Cancer Institute). Whole-exome or whole-genome sequencing was conducted on neurosphere models to obtain copy number alterations.

### Public data sets.

Log_2_(TPM) + 1 RNA sequencing, CERES gene dependency scores, and DNA methylation array data were downloaded from the DepMap portal (https://depmap.org/portal/, CCLE expression: 21Q3) (40). Density plots displaying the distribution of CERES scores per tumor lineage were generated with ggridges software in R (v4.0.3). Projection of *VRK2* or *VRK1* expression for tumor lineages from TCGA/TARGET/Treehouse tumor data sets was generated using uniform manifold approximation and projection plots available in the Celligner alignment portal (https://depmap.org/portal/celligner/). TCGA LGG and GBM gene expression and clinical data were downloaded from https://www.cbioportal.org Methylation data for pediatric high-grade gliomas from Mackay et al. were downloaded from ArrayExpress (https://www.ebi.ac.uk/arrayexpress/experiments/E-MTAB-5528/) (41).

### Lentiviral production.

Lentiviral production was conducted using HEK293T cells, as described on the Broad Institute Genetic Perturbation Platform (GPP) web portal (https://portals.broadinstitute.org/gpp/public/). Briefly, high-titer lentivirus was produced by transfection of HEK293T cells (ATCC catalog CRL-3216) with the lentiviral vector, psPAX2 (Addgene 12260) and vsvg (Addgene 8454) with Lipofectamine 2000 (Life Technologies catalog 11668027). Viral supernatant was collected 48 hours after transfection and filtered with a 0.2 μm filter. Cells were transduced with virus in the presence of 5 μg/mL polybrene (Santa Cruz Biotechnology catalog sc-134220) and selected with blasticidin (5 μg/mL) (Life Technologies catalog R21001) or puromycin (1 μg/mL) (Gibco, Thermo Fisher Scientific, catalog A1113803) according to appropriate selection agent. dTAG-HA-VRK1–expressing cell lines were derived by first expressing stable dTAG-HA-VRK1 prior to infection with sgVRK1#2.

### sgRNAs.

The sgRNA sequences used for the validation experiments were designed using the web-based program CRISPick provided by the Broad Institute GPP (https://portals.broadinstitute.org/gppx/crispick/public). For the CRISPR-mediated gene KO, annealed oligonucleotides carrying the sgRNA target sequence as well as the cloning adapters were inserted into a guide RNA–expressing vector that also expresses a puromycin resistance gene (pXPR_003, Broad Institute GPP), the vector expressing the hygromycin resistance gene (pXPR_016, Broad Institute GPP), or guide vectors with GFP or mCherry coexpression (LCV2_EGFP or LCV2_mCherry). LCV2_EGFP and LCV2_mCherry were gifts from Jason Moffat (University of Toronto, Toronto, Ontario, Canada) (Addgene plasmids 155098 and 155096) (42). The targeting sequences for the individual sgRNAs are outlined in Supplemental Table 2. For tamoxifen-inducible sgRNA expression, we utilized the CRISPR-Switch system as described by Chylinski et al. (17). Guides were cloned into the vector pLenti_Switch-ON, which was a gift from Ulrich Elling (Institute of Molecular Biotechnology, Austrian Academy of Sciences, Vienna, Austria).

### Open reading frame constructs.

Codon-optimized, sgRNA-resistant DNA fragments encoding VRK1^WT^, VRK1^K179E^, and VRK2^WT^ were purchased from gBlock (IDT) and cloned into pDONR-221 via BP gateway cloning. VRK2^K168E^ was generated through the QuickChange II site-directed mutagenesis kit (Agilent Technologies) using the primer 5′-GAATATGTTCATGGTGATATAGAAGCAGCAAATCTAC-3′. BAF^WT^ and its mutants (S4A and S4D) were synthesized with gateway-compatible AttP flanking sites (IDT) and cloned into pDONR-221. Entry clone pENTR/D_creERt2 was a gift from Leonard Zon, Harvard Mecial School (Addgene plasmid 27321) (43). VRK1^WT^ was further cloned into pLEX_305-C-dTAG (Addgene 91798), and VRK1^WT^, VRK1^K179E^, VRK2^WT^, VRK2^K168E^, and Cre-ERT2 were further cloned into pLEX_307 (Addgene 41392) via LR gateway cloning (LR clonase II enzyme mix, Thermo Fisher Scientific, catalog 11791-100). BAF^WT^, BAF^S4A^, and BAF^S4D^ were cloned into doxycycline-inducible expression vector PLXI403 (Addgene 41395).

Cells were transduced with virus in the presence of 5 μg/mL polybrene and selected with blasticidin (5 μg/mL) or puromycin (1 μg/mL). dTAG-HA-VRK1–expressing cell lines were derived by first expressing stable dTAG-HA-VRK1 prior to infection with sgVRK1#2.

Further information about and requests for reagents should be directed to and fulfilled by the lead contact, WCH. Plasmids for the C-terminus; dTAG-VRK1; sgRNAs 1, 2, and 4; and cDNAs for VRK1^WT^, VRK1^K179E^, VRK2^WT^, VRK2^K168E^, BAF^WT^, BAF^S4A^, and BAF^S4D^ will be made available on Addgene.

### Bisulfite sequencing.

Genomic DNA was extracted using a DNeasy Blood & Tissue Kit (Qiagen 69505). DNA was bisulfite-converted using an EpiTect Bisulfite Kit (Qiagen 59104). Bisulfite-converted DNA was PCR-amplified with the EpiMark HotStart Taq (New England Biolabs M0490) using the following primers: VRK2 TSS primer set: forward 5′-TAGGTTGTGGTATAGGAGATTTAATATT-3′, reverse 5′-AATAAAAACTATATTACTACCTCCACCC-3′. PCR was performed at an annealing temperature of 59°C for 40 cycles. PCR products were visualized on 2% E-Gel EX agarose gels (Thermo Fisher Scientific, catalog G401002) for correct size and band patterning. PCR products were then column-purified using the QIAquick PCR Purification Kit and submitted for difficult template Sanger sequencing with Azenta with both the forward and reverse primers.

### Cell proliferation assay.

The viability effect of *VRK1* KO in GBM cell lines and primary DMG neurosphere models was determined by the clonogenic cell proliferation assay. Briefly, cells were transduced with guide RNA sgCtrl or sgVRK1. Following 1 week under selection, 0.5 × 10^4^ to 1 × 10^4^ cells per well were seeded in 6-well Falcon plates (Thermo Fisher Scientific, catalog 087721B) in triplicate. The medium was changed every 5–7 days. After 7–10 days, cell numbers were counted using the Vi-Cell automated cell counter (Beckman Coulter, catalog 731196).

For viability effect in NB cell lines, 5 × 10^5^ cells of sgChr2, sgVRK1#1, sgVRK1#2, or sgVRK1#4 were plated onto 6 cm dishes. For dTAG-VRK1 cells, 5 × 10^5^ cells were plated and attached 16 hours prior to incubation with either DMSO vehicle or 1 μM dTAG^V^-1. After 2–3 days, cells were detached and counted, and the number of doublings relative to the prior time point was calculated. Groups were replated at 5 × 10^5^ cells per group, and the same steps were repeated every 2–3 days for a total of 14 days. For days in which fewer than 5 × 10^5^ cells were counted, all the cells were plated. Population doublings were calculated by the total cells compared with the number of seeded cells. Values were added to the previous time point, starting at 0 for day 0. dTAG-VRK1 cells remained in vehicle or 1 μM dTAG^V^-1 for the entirety of the 14 days.

For crystal violet staining, cells were plated in 6-well plates and stained/fixed with 2.5 mg/mL solution of crystal violet (MilliporeSigma, catalog C3886) in 20% methanol.

### Cell cycle and apoptosis assay by flow cytometry.

Cells were harvested, washed, and fixed in ice-cold 70% ethanol and then resuspended in stain buffer containing PI and RNase (BD, catalog 550825). Apoptosis was assessed using annexin V and PI staining according to the manufacturer’s instructions (Invitrogen, catalog 88-8005-74). Samples were analyzed on a BD LSRII flow cytometer. Data analysis was completed using the cell cycle analysis package in FlowJo ver.10.8.0 (Tree Star).

### Western blot.

Cell pellets were lysed with Cell Signaling Technology lysis buffer (catalog 9803) that was supplemented with phosphatase inhibitor (Roche, catalog 04906845001) and protease inhibitor (Roche, catalog 11836170001) and diluted to 1 μg/μL in sample buffer.

Approximately 35 μg of whole-cell lysate protein was loaded into wells and resolved in 4%–12% acrylamide gradient gels. Whole-cell lysates were run with MOPS running buffer solution (Thermo Fisher Scientific, catalog NP0001) for high–molecular weight proteins and MES running buffer solution (Thermo Fisher Scientific, catalog NP0002) for low–molecular weight proteins. Acrylamide gels were wet-transferred onto nitrocellulose or PVDF membranes for at least 90 minutes. Primary antibodies listed in Supplemental Table 3 were diluted in 3% BSA in TBS-Tween and incubated overnight at 4°C. Rabbit polyclonal anti–phospho-BAF antibody was a gift from Robert Craigie (NIH, Bethesda, Maryland, USA). Secondary goat anti-rabbit IRDye 800 (LICOR, catalog 926-32211) or goat anti-mouse IRDye 680 (LICOR, catalog 926-68070) antibodies were diluted at 1:5,000 in TBS-Tween and incubated at room temperature for 1 hour. All membranes were imaged on LICOR Odyssey infrared imaging system at 680 and 800 nm wavelengths and analyzed with ImageStudio Odyssey Lite Software (LICOR).

### IncuCyte CASP3/7 assay.

LN443-Cas9 cells were transduced with guide RNA sgCtrl or sgVRK1. Following 1 week under selection, 5 × 10^4^ cells per well were seeded in 24-well Falcon plates (Thermo Fisher Scientific, catalog 353047). Then 5 mM of IncuCyte Caspase-3/7 Green Apoptosis Assay Reagent (Sartorius, catalog 4440) as well as 1:500 of Nuclight Rapid Red Dye (Sartorius, catalog 4717) were added to each well. The plate was transferred into the IncuCyte S3 Live-Cell Analysis System (Sartorius, catalog 4647) for imaging. Phase contrast images and green/red fluorescence channel images were captured using the 10× objective magnification every 4 hours for a total of 48 hours. For each well, 4 images containing both phase contrast and green channel data were obtained.

Using the IncuCyte S3 Analysis System software, cell confluence over time was quantified along with the intensity of green (apoptosis-positive) objects in mm^2^/well. Computer-generated masks for confluence and green area, trained on a sample set of images across time points and confluence levels, were manually checked for accuracy. Each metric was averaged over the 4 quadrants per well. First, the green object total intensity metric for each well was divided by the confluence metric for each well, yielding a normalized measure of caspase-3/7 activity.

### Mass spectrometry sample preparation.

Samples were processed with the streamlined TMT protocol and phospho-enrichment methods described previously (44). Data were acquired with Orbitrap Eclipse mass spectrometer with FAIMS and coupled to a Proxeon NanoLC-1200 UHPLC (Thermo Fisher Scientific). The mass spectrometry proteomics data have been deposited to the ProteomeXchange Consortium via the PRIDE (45) partner repository with the data set identifier PXD030599.

### Mass spectrometry data analysis.

A suite of in-house software tools were used for.RAW file processing, controlling peptide- and protein-level false discovery rates, assembling proteins from peptides, and quantifying protein from peptides as previously described (46). MS/MS spectra were searched against a UNIPROT Human database with both the forward and reverse sequences. Database search criteria are as follows: tryptic with 2 missed cleavages, a precursor mass tolerance of 50 ppm, fragment bin tolerance of 0.02, static alkylation of cysteine (57.02146 Da), static TMT labeling of lysine residues and N-termini of peptides (304.2071 Da), variable oxidation of methionine (15.99491 Da), and variable phosphorylation on serine, threonine, and tyrosine (+79.966 Da). Phosphorylation site localization was determined using the AScore algorithm (47) using a threshold of 13 corresponding to 95% confidence in site localization. TMT reporter ion intensities were measured using a 0.003 Da window around the theoretical *m/z* for each reporter ion. Proteins with <100 summed signal-to-noise across all channels and <0.5 precursor isolation specificity were excluded from the final data set.

Ratios were calculated between peptide quantitation at 4 hours after dTAG^V^-1 versus 0 hours and at 8 hours after dTAG^V^-1 versus 0 hours. *P* values for each ratio were calculated using Student’s 2-tailed *t* test. From the fold change values and *P* values, KSEA (https://casecpb.shinyapps.io/ksea/) was performed with NetworKIN score cutoff of 3 (14, 48–50).

### Immunofluorescence.

The nuclear membrane and DNA damage foci were visualized by immunofluorescence using the following procedure. LN443-Cas9, SF172-Cas9, NB-1, and Kelly cells were transduced with various sgRNAs. BAF and Emerin were imaged by transducing GFP-tagged constructs. EGFP-BAF was a gift from Daniel Gerlich (Institute of Molecular Biotechnology, Austrian Academy of Sciences, Vienna, Austria) (Addgene plasmid 101772) (51). pLVX-EF1a-EGFP-Emerin-IRES-Hygromycin was a gift from David Andrews (University of Toronto, Toronto, Ontario, Canada) (Addgene plasmid 134864) (52). For the BAF experiment, inducible BAF wild-type or mutant expression vectors were transduced in LN443 cells. Following selection (~5–7 days), doxycycline induction (0.5 μM for 3 days), or dTAG^V^-1 treatment (0.5 μM for 1 day), cells were seeded onto 1½ cover glasses (MilliporeSigma, catalog CLS285018) in 6-well Falcon plates (Thermo Fisher Scientific, catalog 087721B). The next day, cells were fixed with 4% formaldehyde (VWR, catalog 100503) diluted in PBS. Fixed cells were permeabilized and blocked with 0.1% Triton-X in 50% Odyssey Blocking Buffer (LICOR, catalog 927-70001) in PBS for 1 hour at room temperature. The cells were then incubated with the primary antibody at the specified dilution in 0.1% Triton-X with 50% Odyssey Blocking Buffer, overnight at 4^o^C. After washing 3 times with PBS, cells were incubated with the secondary antibody at the specified dilution in 0.1% Triton-X with 50% Odyssey Blocking Buffer for 1 hour at room temperature. The cells were then washed 3 times with PBS and mounted onto glass slides with ProLong Gold antifade mounting medium with DAPI (Life Technologies, catalog P36941). Imaging was conducted using an Olympus IX73 inverted microscope, an Olympus DP80 charge-coupled device camera, and 20×/40×/100× objectives. Antibodies used are listed in Supplemental Table 3.

### Live-cell imaging.

dTAG-VRK1-LN443 or dTAG-VRK1-NB-1 cells were transduced with EGFP-BAF as previously described. A total of 2 × 10^4^ to 5 × 10^4^ per well were then seeded in MatTek 24-well, glass-bottom plates (Thermo Fisher Scientific NC1284979). At 4 hours following dTAG^V^-1 addition, the plate was imaged using the 40× objective in a Leica DMi8 Widefield microscope with automated stage, an Oko-Lab stage-top incubator, and Oko-Lab CO_2_/humidity controller. Every 20 minutes for 48 hours, 3 × 3 fields per well were imaged. Image stitching was performed using the Leica LAS X software platform. Subsequent image analysis was performed using ImageJ ver. 1.53m (NIH).

### In vivo tamoxifen-inducible sgRNA xenografts.

This study was approved by the Institutional Animal Care and Use Committee (IACUC) of Dana-Farber Cancer Institute and performed under protocol 04-101. IACUC guidelines on the ethical use and care of animals were followed. SF295 cells constitutively expressing Cas9 were infected with tamoxifen-inducible sgRNAs targeting Chr2-2 or *VRK1*. A total of 6 × 10^6^ cells were resuspended in 1:1 vol/vol Matrigel/media and subcutaneously implanted into the left and right fat pads of 6- to-8-week-old female NSG mice (The Jackson Laboratory stock 005557). When either tumor was about 100–200 mm^3^ mice were randomized to tamoxifen or vehicle treatment. Tamoxifen was delivered by 3 daily intraperitoneal injections of approximately 3 mg. Tamoxifen (MilliporeSigma) was prepared at a stock concentration of 30 mg/mL in corn oil. The control group received an equal volume of corn oil. Tumors were measured by Vernier caliper, and volume was determined using the standard formula [(length × width^2^)/2 where length is always the larger measurement]. Animals were euthanized once they reached a humane endpoint, and tumor tissue was flash-frozen or formalin-fixed for later protein extraction. All mice that developed tumors were included in the analysis.

At 7 days following treatment with tamoxifen or vehicle control, tumors were collected from a subset of xenografted mice. These were fixed in formalin and embedded in paraffin. Immunohistochemistry was performed following standard protocol, staining for phospho-H2AX (S139) (Cell Signaling Technology catalog 9718; 1:500).

### In vivo doxycycline-inducible sgRNA xenografts.

This study was approved by the IACUC of Dana-Farber Cancer Institute and performed under protocol 04-101. IACUC guidelines on the ethical use and care of animals were followed. Kelly cells constitutively expressing Cas9 were infected with doxycycline-inducible sgRNAs targeting Chr2-2 or *VRK1*. A total of 4 × 10^6^ cells were resuspended in 1:1 vol/vol Matrigel/media and subcutaneously implanted into the left and right fat pads of 6- to-8-week-old female NSG mice (The Jackson Laboratory stock 005557). When either tumor was approximately 50 mm^3^ mice were randomized to doxycycline-containing (625 parts per million) or regular diet. Tumors were measured by Vernier caliper, and volume was determined using the standard formula [(length × width^2^)/2 where length is always the larger measurement]. Animals were euthanized once they reached a humane endpoint, and tumor tissue was flash-frozen or formalin-fixed for later protein extraction. All mice that developed tumors were included in the analysis.

### Intracranial xenografts.

This study was approved by the IACUC of Dana-Farber Cancer Institute and performed under protocol 18-006. IACUC guidelines on the ethical use and care of animals were followed. Intracranial xenografts were established with the patient-derived neurosphere line SU-DIPGXIIIP*Luci, with doxycycline-inducible guides. Cells were injected stereotactically into the striatum of 6-week-old female NSG mice treated with buprenorphine 0.05 mg/kg and anesthetized with isoflurane 2%–3%. The skull of the mouse was exposed through a small skin incision, and a small burr hole was made using a drill at the selected stereotactic coordinates zeroed on bregma: –2.5 mm *x*, –1 mm *y*, and –3.0 mm *Z*. The cells (100,000 cells in 1 μL PBS per mouse) were injected using a 26-gauge Hamilton syringe. After we closed their scalp with suture and staple, mice were returned to their cages, placed on a warming pad, and visually monitored until full recovery. The same day following the procedure, CRISPR guide expression was induced through doxycycline chow. Mice were then checked daily for signs of distress, including seizures, weight loss, and tremors, and euthanized as they developed neurological symptoms, including head tilt, seizures, sudden weight loss, loss of balance, and/or ataxia.

Tumor growth was monitored every 1–2 weeks using the Spectrum In Vivo Imaging System (PerkinElmer). Briefly, mice were injected intraperitoneally with 75 mg/kg d-luciferin potassium salt (Promega E1605) in sterile PBS, then anesthetized with 2% isoflurane in medical air. Serial bioluminescence images were acquired using the automated exposure setup. The peak bioluminescence signal intensity within selected regions of interest was quantified using the Living Image Software (PerkinElmer), expressed as photon flux (p/s/cm^2^/sr). Representative planar bioluminescence images were displayed with indicated adjusted minimal and maximal thresholds.

### Data availability.

The mass spectrometry proteomics data have been deposited to the ProteomeXchange Consortium via the PRIDE (45) partner repository with the data set identifier PXD030599.

### Statistics.

For statistical tests of significance, the statistical test and *P* values are described in the respective figure legends. All *t* tests are 2-sided unless otherwise indicated. A *P* value of 0.05 was used as the cutoff for significance unless otherwise indicated. These values were calculated in GraphPad Prism (version 9.3.0 for Windows, GraphPad Software) or R version 4.0.2 and Rstudio version 1.2.5042. Error bars represent SD unless otherwise indicated. All duplicate measures were taken from distinct samples rather than repeated measures of the same sample.

### Study approval.

This study was approved by the IACUC of Dana-Farber Cancer Institute and performed under protocols 04-101 and 18-006.

## Author contributions

Conceptualization was done by JS, NWM, BE, KHC, MGF, KS, and WCH. Data curation was done by JS, NWM, and BE. Formal analysis was done by JS, NWM, BE, KHC, SMM, and BS. Investigation was done by JS, NWM, BE, KHC, SMM, SY, MCT, JGM, JJK, BS, SP, EP, TQ, DAS, DJ, DLM, and JH. Writing of the original draft was done by JS, NWM, BE, MGF, KS, and WCH. Resources were provided by RB, KLL, KS, MGF, and WCH. Supervision was provided by RB, KLL, KS, MGF, and WCH. Funding was provided by RB, KLL, KS, MGF, and WCH. Co–first authors were ordered by experimental and draft writing contribution.

## Supplementary Material

Supplemental data

Supplemental video 1

## Version 1 08/30/2022

In-Press Preview

## Version 2 10/10/2022

Electronic publication
