# Supplementary material for: VRK1 as a synthetic lethal target in VRK2 promoter–methylated cancers of the nervous system
Source: JCI Insight. 2022 Oct 10;7(19):e158755. doi: 10.1172/jci.insight.158755 (PMC9675470; doi:10.1172/jci.insight.158755)
Supplement: Supplemental data [file jciinsight-7-158755-s017.pdf]

Supplementary Figure 1.

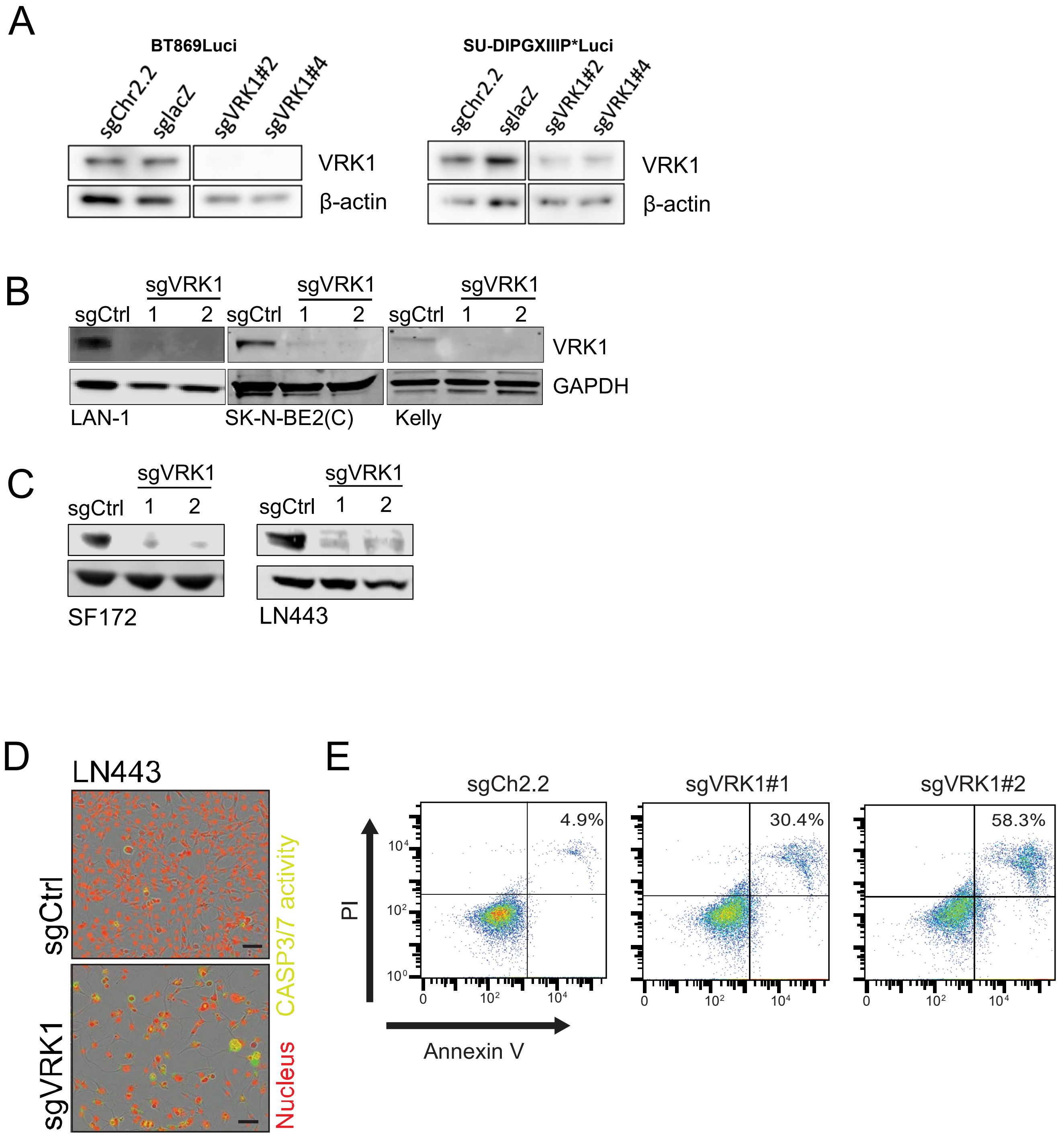

Supplementary Figure 2.

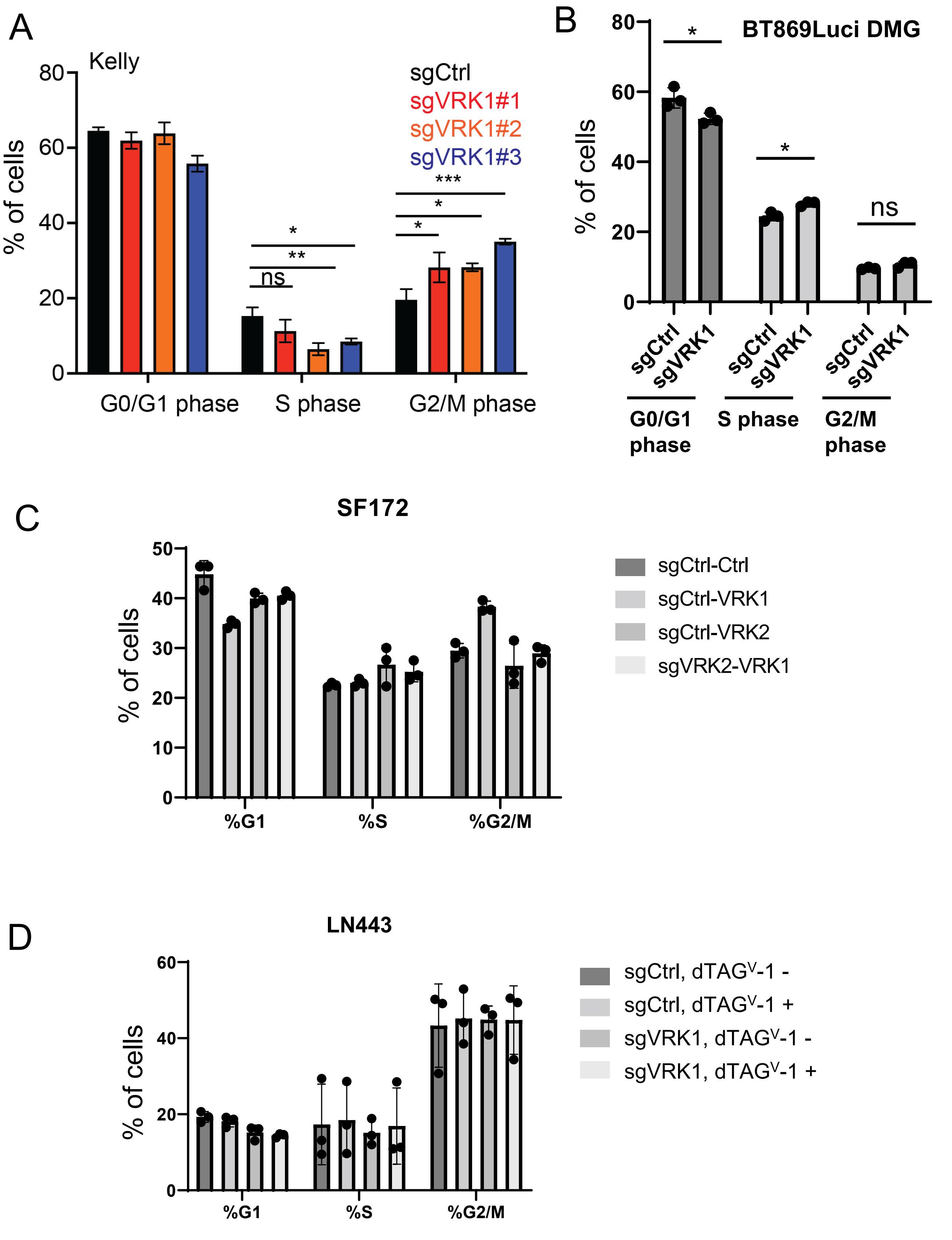

Supplementary Figure 3.

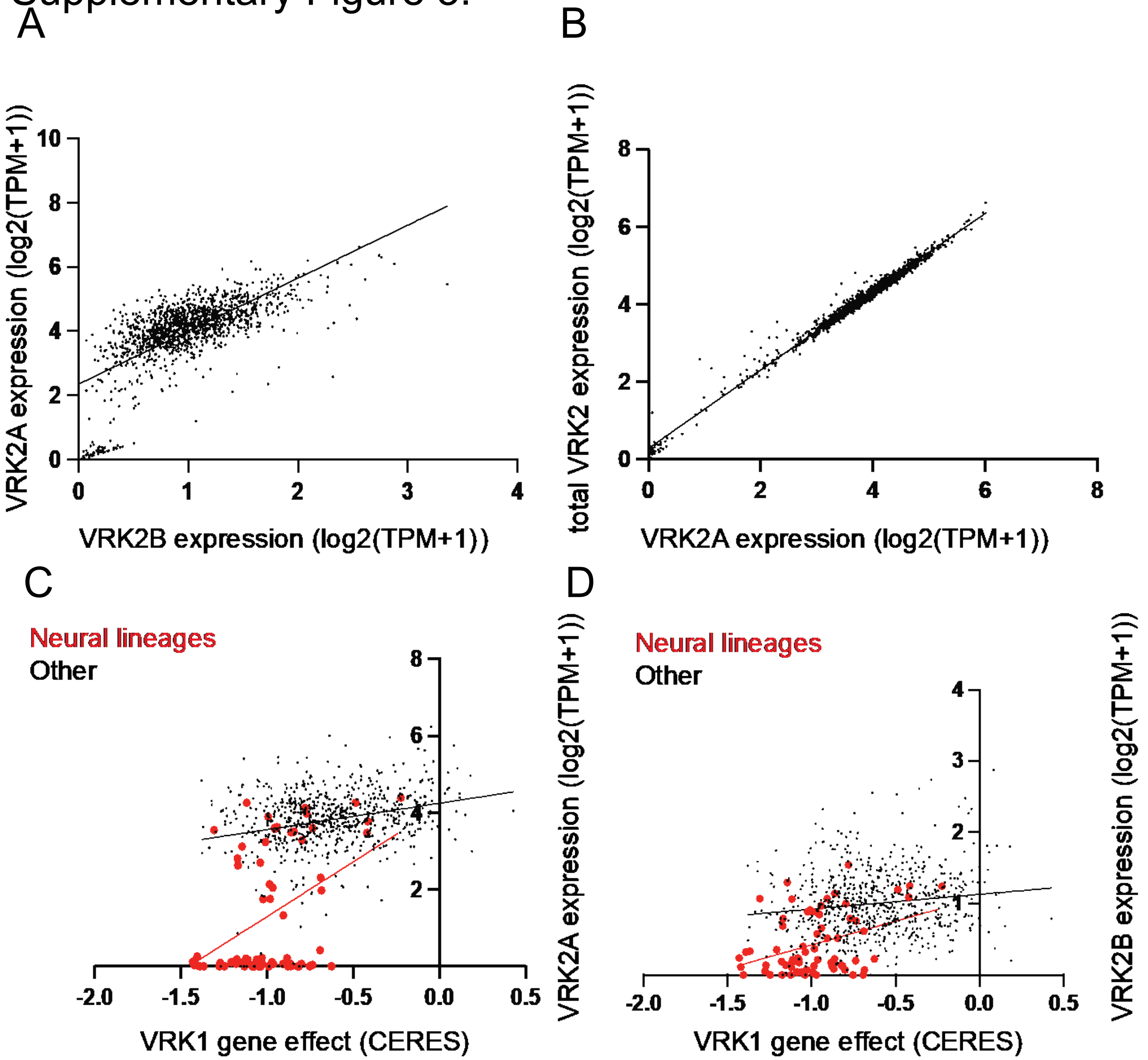

Supplementary Figure 4.

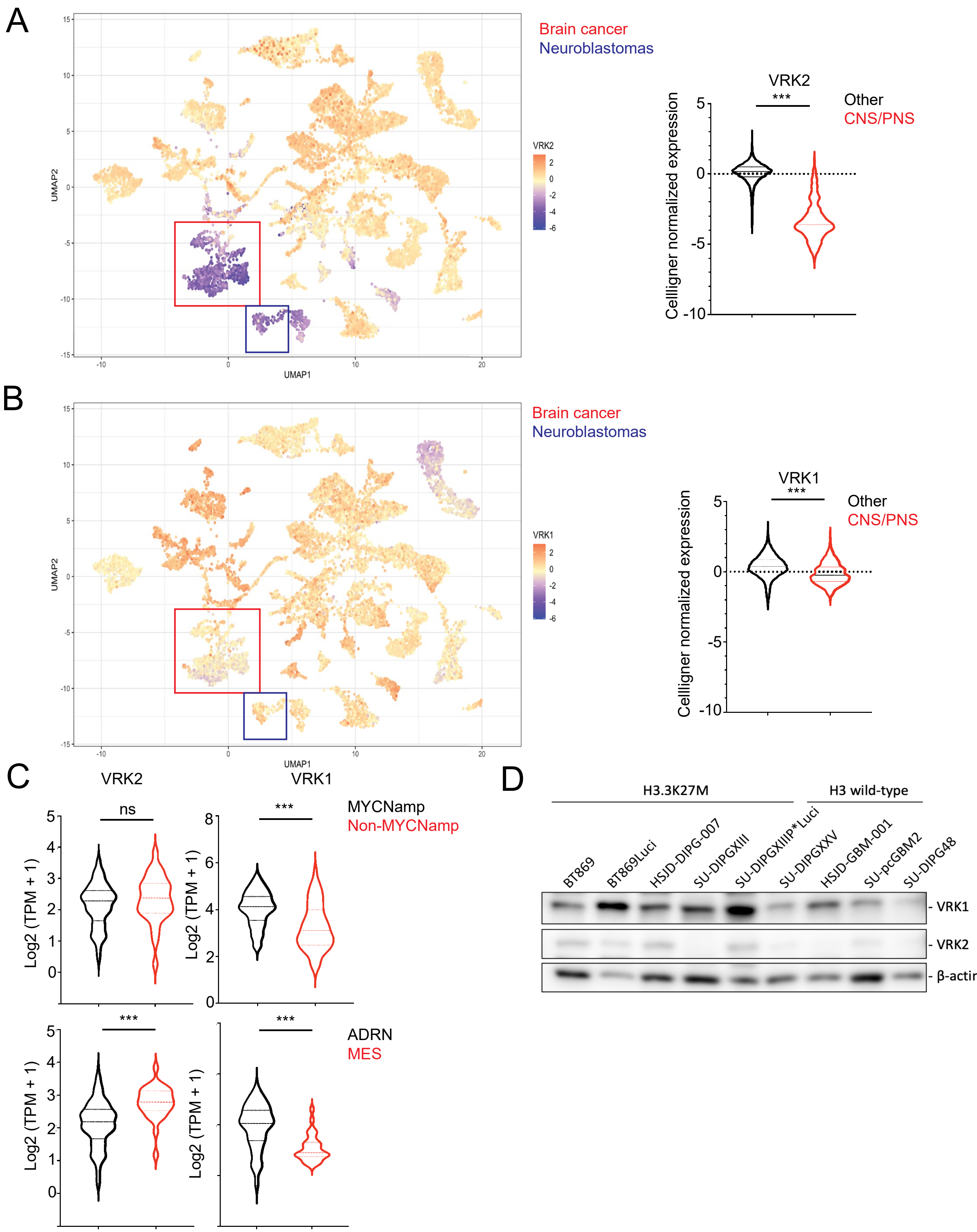

Supplementary Figure 5.

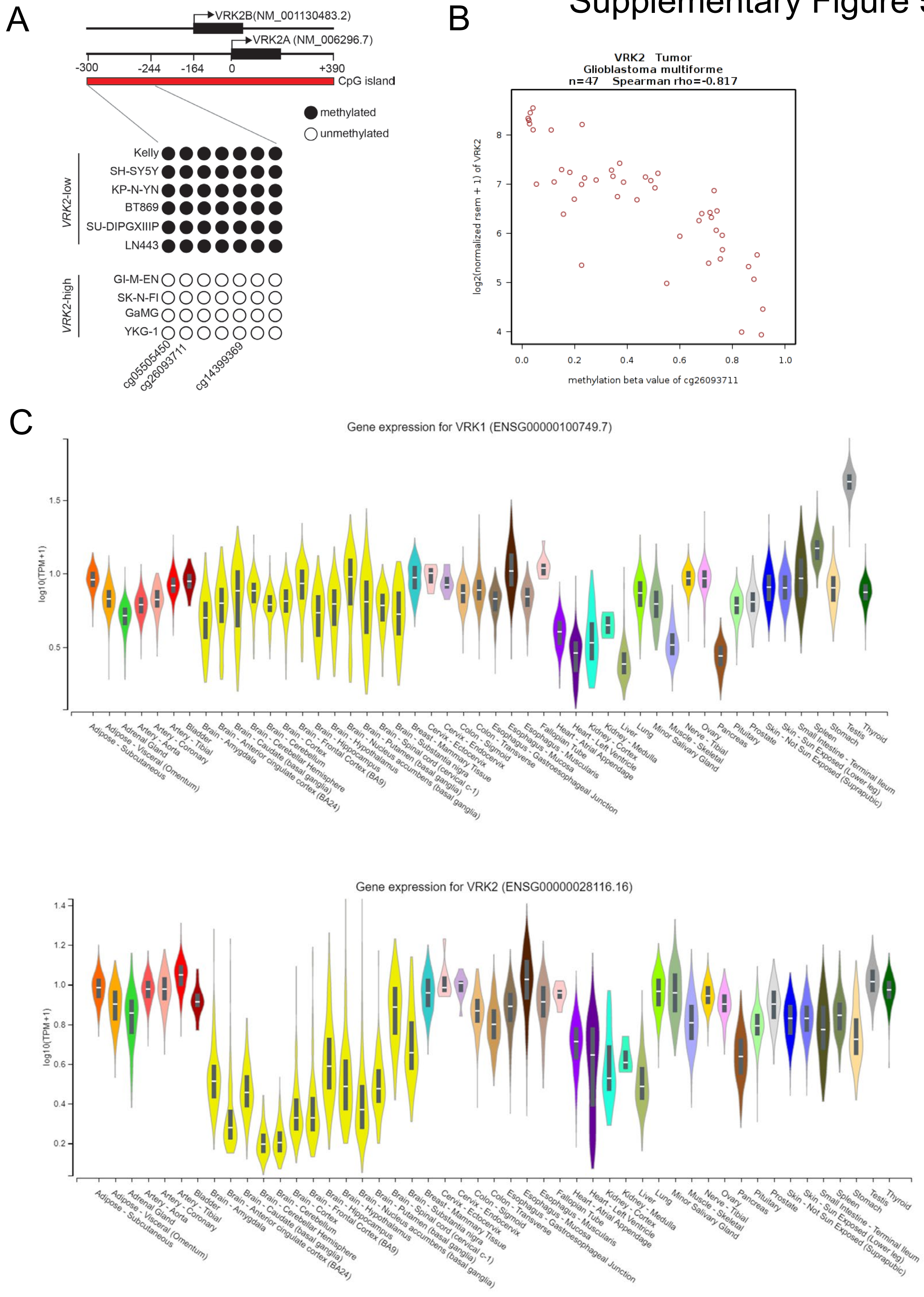

Supplementary Figure 6.

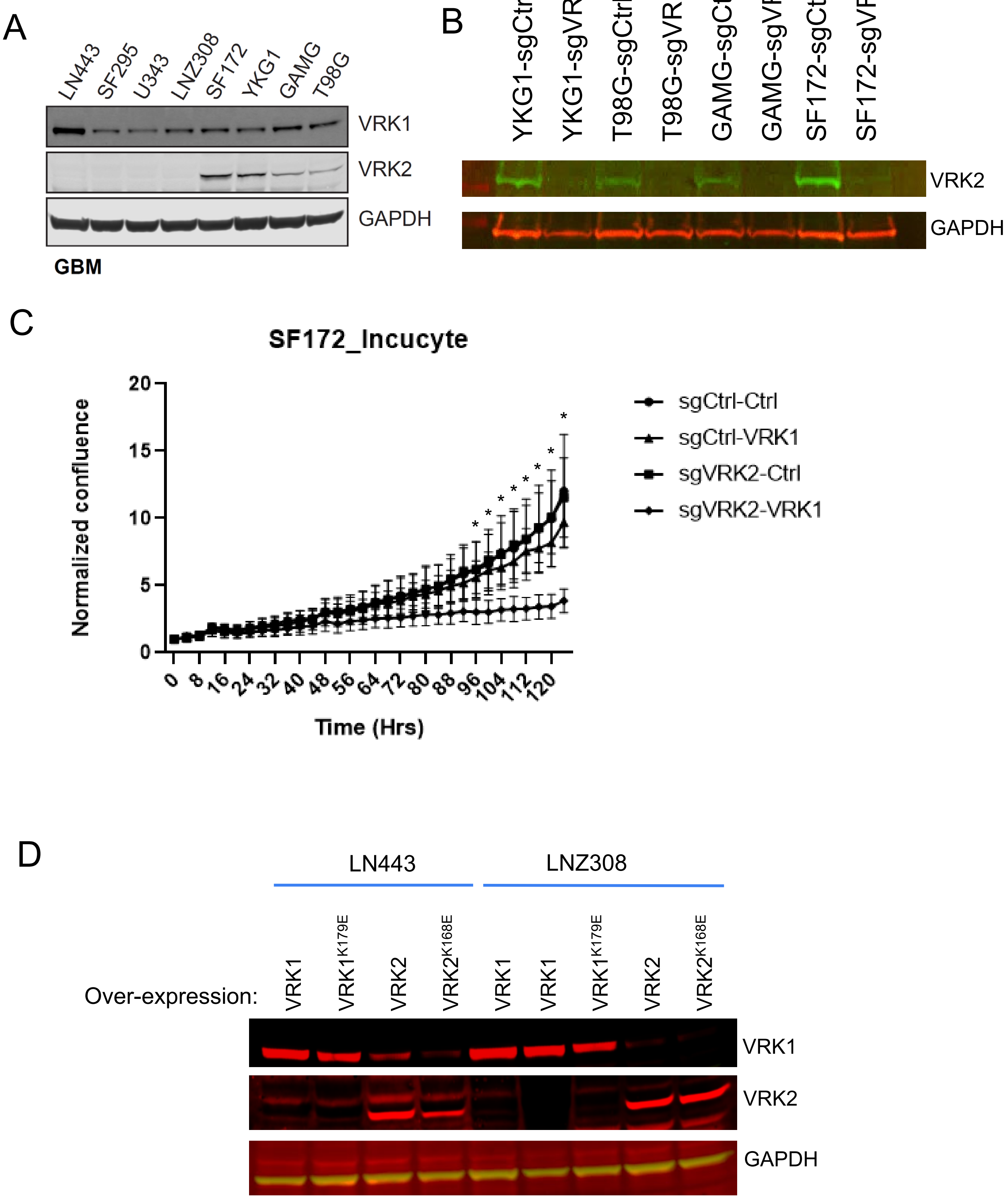

Supplementary Figure 6 cont.

E

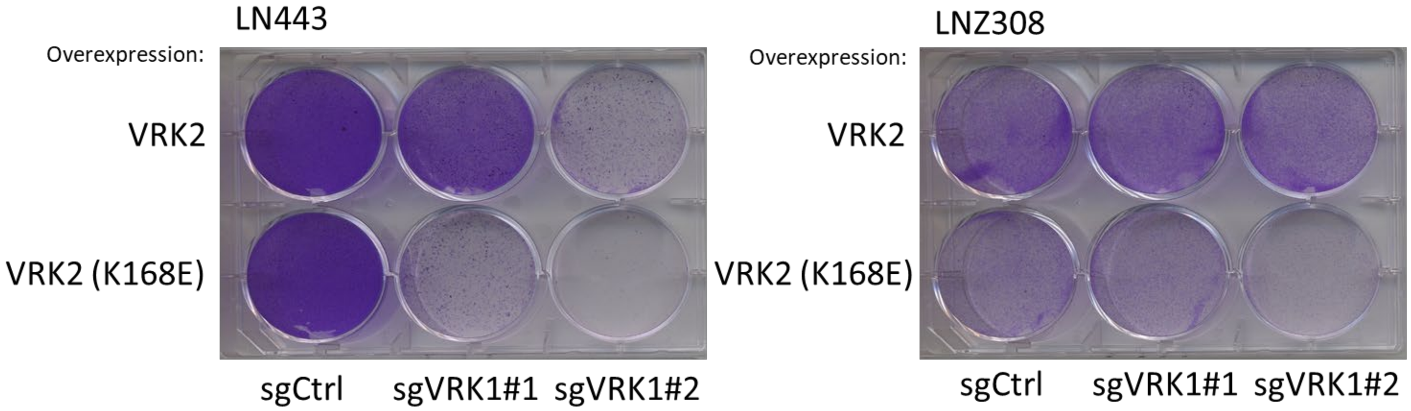

F

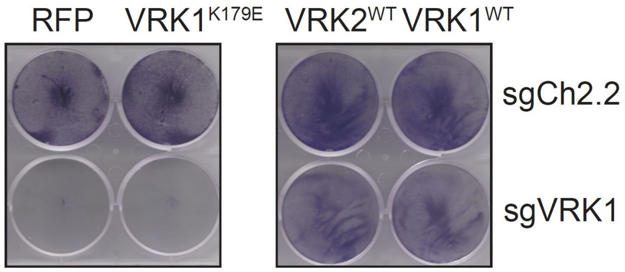

G

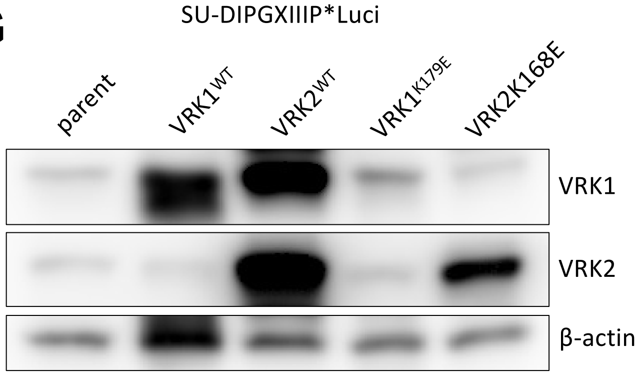

H

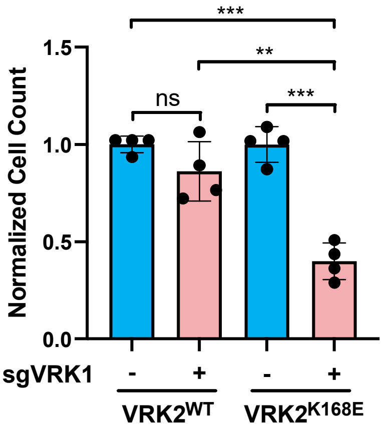

I

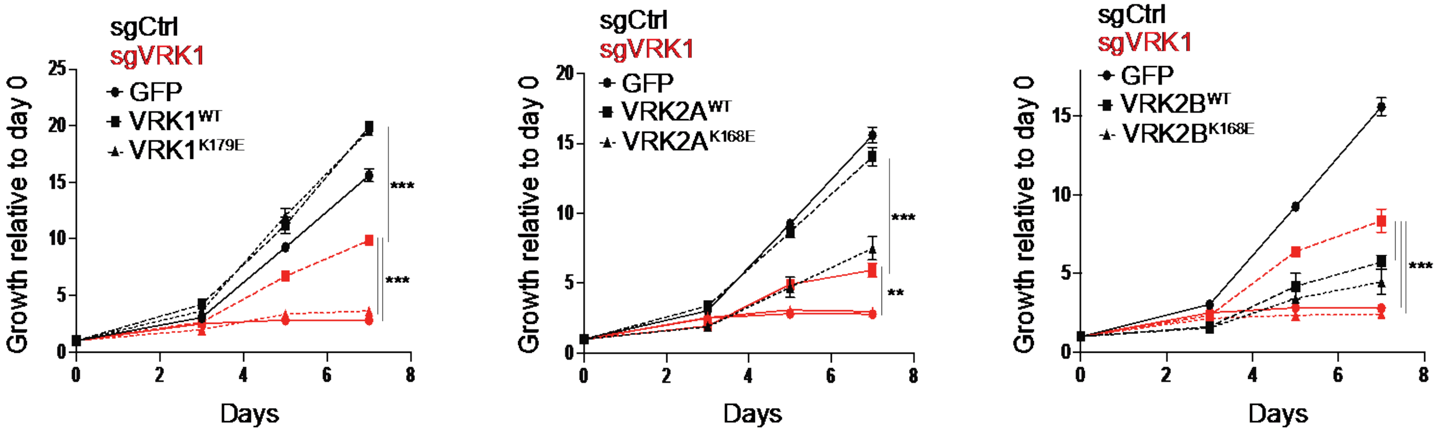

Supplementary Figure 7.

A

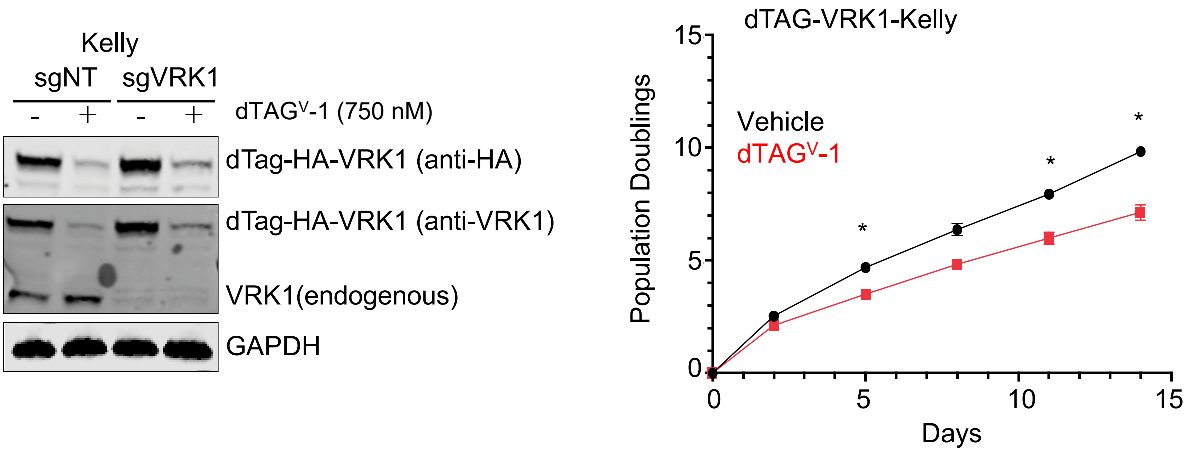

B

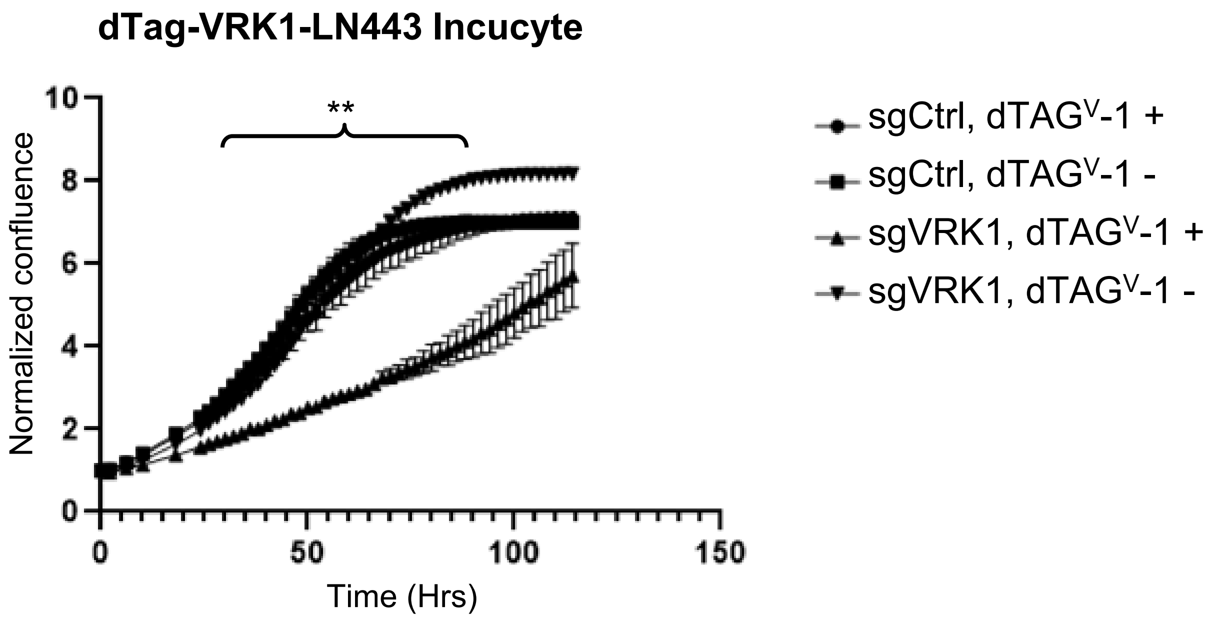

Supplementary Figure 8.

A

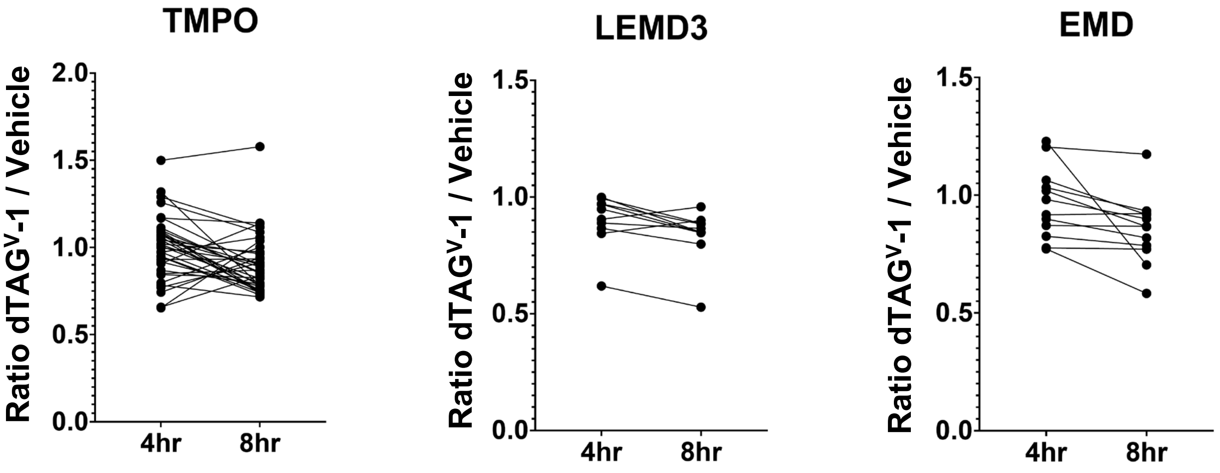

Supplementary Figure 9.

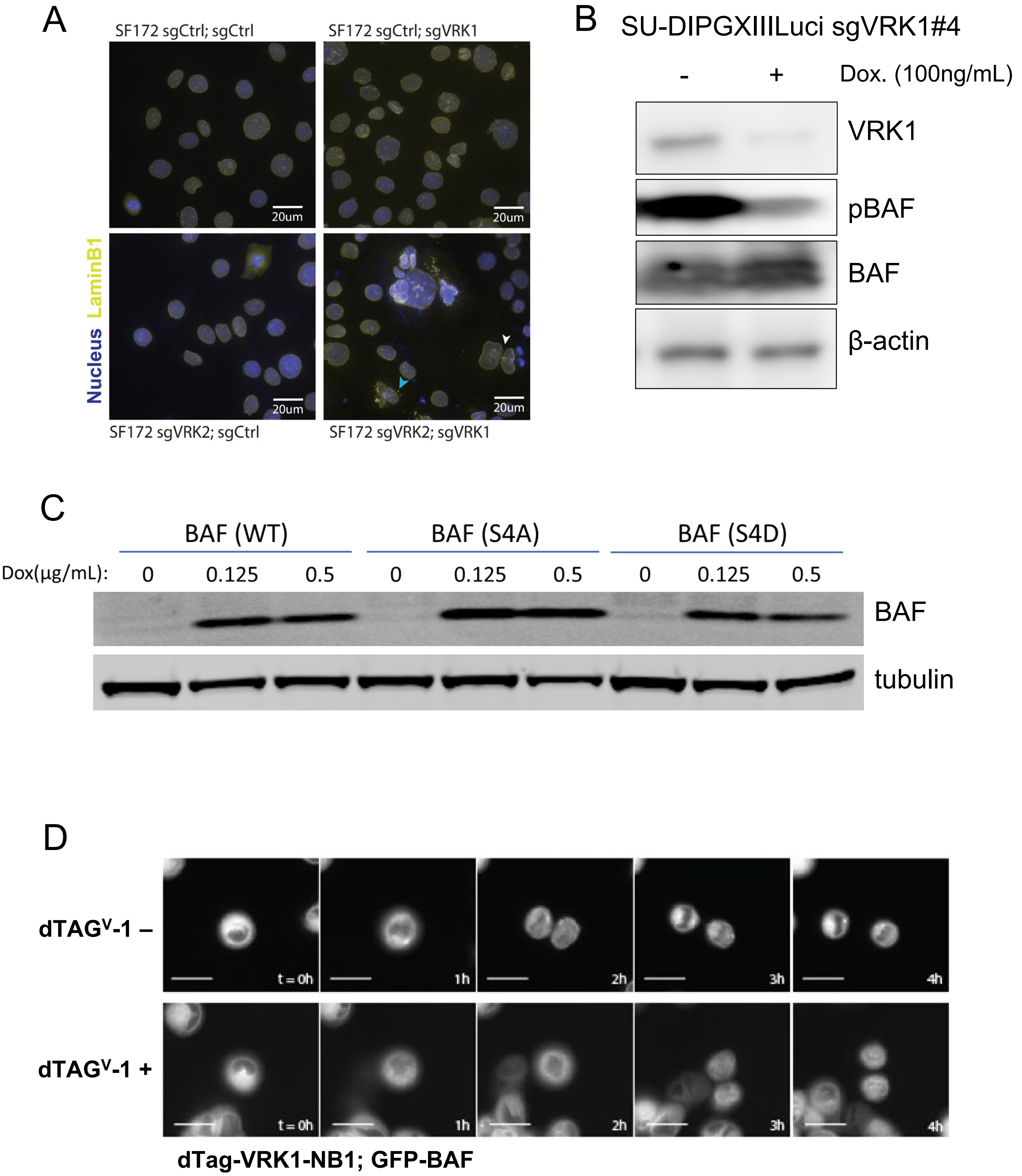

Supplementary Figure 10.

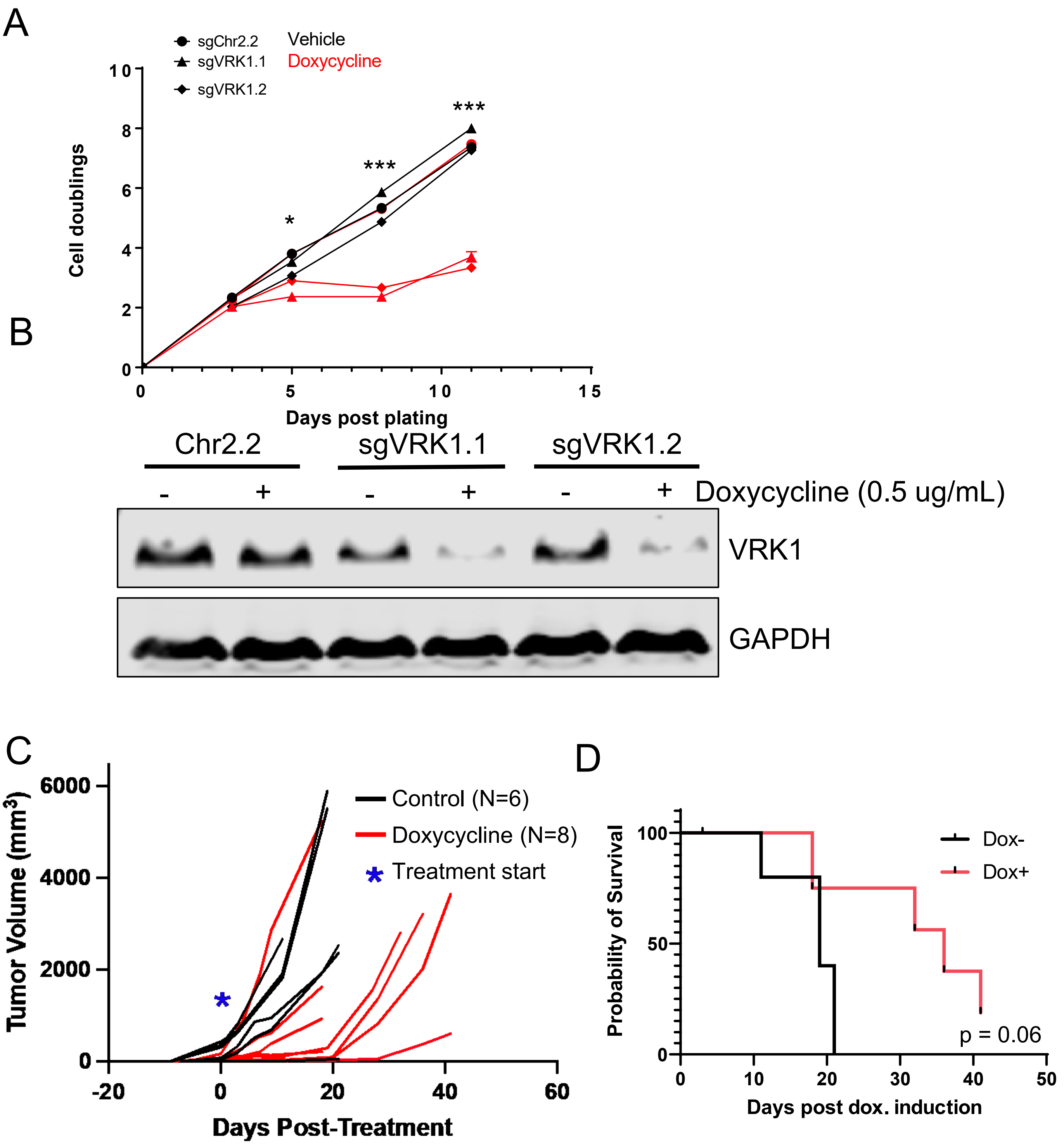

Supplementary Table 1

A) Neuroblastoma cell line models

Cell line	Cell state	Hotspot ALK mutation?	Hotspot NRAS mutation?	Hotspot TP53 mutation?	MYCN amplification?
CHLA15	ADRN	Y	N	N	N
CHP212	MES	N	Y	N	Y
COGN278	ADRN	N	N	N	Y
COGN305	ADRN	N	N	N	Y
GIMEN	MES	N	N	N	N
IMR32	ADRN	N	N	N	Y
KELLY*	ADRN	Y	N	Y	Y
KPNYN	ADRN	N	N	N	Y
LAN-1	ADRN	Y	N	Y	Y
LAN2	ADRN	N	N	Y	Y
LS	ADRN	N	N	N	Y
MHHNB11	ADRN	N	N	N	Y
NB1*	ADRN	N	N	N	Y
NB1643	ADRN	Y	N	N	Y
NGP	ADRN	N	N	Y	Y
SIMA	ADRN	N	N	N	Y
SKNAS	MES	N	Y	N	N
SKNBE2	ADRN	N	N	Y	Y
SKNBE2C*	ADRN	N	N	Y	Y
SKNDZ	ADRN	N	N	Y	Y
SKNFI	ADRN	N	N	Y	N
TGW	ADRN	Y	N	Y	Y

* validated in paper

B) Pediatric GBM and DMG patient-derived neurosphere models

Model	Histology	Histone H3 mutation	Hotspot ACVR1 mutation?	Hotspot TP53 mutation?
BT869*	DMG	H3.3 K27M	Y	N
HSJD-DIPG-007	DMG	H3.3 K27M	N	N
HSJD-GBM-001	GBM	WT	N	N
SU-DIPG-XIII*	DMG	H3.3 K27M	N	N
SU-DIPG-XXV	DMG	H3.3 K27M	N	N
SU-DIPG-48	DMG	WT	N	N
SU-pcGBM2	GBM	WT	N	N

* validated in paper

Supplementary Table 1 continued

C) GBM cell line models

Cell line	Hotspot IDH1/2 mutation?	Hotspot TP53 mutation?	MGMT methylation?
A1207	N	N	N
A172	N	N	Y
AM38	N	N	Y
CAS1	N	Y	Y
DBTRG05MG	N	N	Y
DKMG	N	N	Y
GAMG*	N	Y	Y
GB1	N	Y	Y
GI1	N	Y	Y
GMS10	N	Y	Y
GOS3	N	N	Y
KALS1	N	Y	N
KNS42	N	Y	Y
KNS60	N	Y	N
KNS81	N	N	Y
LN18	N	Y	N
LN229	N	N	Y
LN340	N	Y	N
LN382	N	Y	N
LN443*	N	Y	Y
LNZ308*	N	N	Y
M059K	N	Y	Y
NMCG1	N	N	Y
SF172*	N	Y	N
SF295	N	Y	Y
SNU1105	N	Y	Y
SNU201	N	N	Y
SNU466	N	N	N
SNU489	N	N	N
SNU626	N	Y	N
T98G	N	Y	N
U178	N	Y	Y
U343	N	N	Y
YH13	N	Y	N
YKG1	N	Y	N

* validated in paper

Supplemental Table 2. CRISPR guide sequences used

sgRNA ID	Target sequence	Note
sgCh2-2	GGTGTGCGTATGAAGCAGTG	A CRISPR cutting control targeting a chromosome 2 intergenic region
sgCtrl	GTGAACCGCATCGAGCTGAA	A control guide targeting EGFP
sgLacZ	AACGGCGGATTGACCGTAAT	A control guide targeting LacZ
sgVRK2	CCTGCAATTAGGTATCCGAA	
sgVRK1#1	CCCAATACTTAGGAACACCC	
sgVRK1#2	GTAGGATTACCCATTGGCCA	
sgVRK1#3	TATATGAAGCAAATGCCAAA	
sgVRK1#4	TGGAAAGTAGGATTACCCAT	

Supplemental Table 3. List of antibodies

Antibody	Dilution	Experiment	Source
Phospho-histone H2AX (S139)	1:100	IF	Thermo Fisher Scientific catalog 05636MI
Phospho-ATR (S428)	1:100	IF	Cell Signaling Technology catalog 2853S
Phospho-DNAPK (S2056)	1:100	IF	Life Technologies catalog PA578130
LaminB1	1:100	IF	Abcam catalog ab16048
Goat anti-rabbit Alexa Fluor 488	1:300	IF	Life Technologies catalog A11008
Goat anti-mouse Alexa Fluor 594	1:300	IF	Life Technologies catalog A32742
Goat anti-rabbit Alexa Fluor 647	1:300	IF	Life Technologies catalog A32733
VRK1	1:1,000	WB	Cell Signaling Technology catalog 3307
VRK2	1:500	WB	Life Technologies catalog MA427456
BAF	1:500	WB	Life Technologies catalog MA534813
Phospho-BAF (S4)	1:1,000	WB	Gift from Robert Craigie (NIH, Bethesda, Maryland, USA)
GAPDH	1:2,000	WB	Cell Signaling Technology catalog 2118
HA	1:1,000	WB	Cell Signaling Technology catalog 2367
β-Actin	1:1,000	WB	Cell Signaling Technology catalog 3700

IF, immunofluorescence; WB, Western blot.

Supplementary Figure Legends

Supplementary figure 1. Validation of *VRK1* CRISPR KO sgRNAs in GBM, NB, and DMG models

A. Immunoblot of VRK1 protein expression following expression of 2 different sgRNAs in the DMG cell lines BT869Luci and SU-DIPGXIIIP*Luci. sgChr2.2 and sgLacZ served as cutting and non-cutting controls, respectively.

B. Immunoblot showing VRK1 expression in LAN-1, SK-N-BE(2)C, or Kelly cell lines with sgRNAs targeting either sgCtrl or *VRK1*.

C. Immunoblot showing VRK1 expression in SF172 or LN443 GBM cell lines with sgRNAs targeting either sgCtrl or *VRK1*.

D. Representative images from live-cell experiment 8 days following infection with sgVRK1 or sgCtrl guide in LN443 GBM cells. Red: nuclear stain; Green: CASP3/7 activity (Incucyte Caspase-3/7 dye).

E. Representative flow cytometry gating strategy for Propidium Iodide and Annexin-V staining following VRK1 KO in NB-1 cells.

Supplementary Figure 2. Cell cycle distribution of GBM, NB, and DMG models following VRK1 depletion

A. Cell cycle distribution of Kelly cells 7 days following *VRK1* KO in three independent sgRNAs (n=3). Significance was determined by one-way ANOVA and Tukey's post-hoc test within each phase of cell cycle. * $p < 0.05$, **$p < 0.001$, ***$p < 0.0001$ ns = not significant.

B. Cell cycle distribution of BT869Luci DMG cells 7 days following *VRK1* KO. Significance was determined by Student's T-test within each phase of the cell cycle. * $p < 0.05$ ns= not significant.

C. Cell cycle distribution of SF172 GBM cells 7 days following combinations of sgCtrl/Ctrl, sgCtrl/VRK1, sgVRK2/Ctrl, or sgVRK2/VRK1 guides. Cell cycle determined by propidium iodide staining, with analysis by FlowJo (ver.10.8.0).

D. Cell cycle distribution of LN443 GBM cells 7 days following *VRK1* KO and degradation of exogenous VRK1 by dTAG^V-1. Cell cycle determined by propidium iodide staining, with analysis by FlowJo (ver.10.8.0).

Supplementary Figure 3. VRK2 isoform expression in cancer cell line models.

A. Scatterplot showing the correlation of VRK2A and VRK2B RNA expression in cancer cell lines found in CCLE. Line denotes linear regression line. R^2=0.46 ; $p < 0.001$

B. Scatterplot showing the correlation of VRK2A and total VRK2 RNA expression in cancer cell lines found in CCLE. Line denotes linear regression line. R^2=0.98 ; $p < 0.001$

C-D. Scatterplots showing the correlation of VRK2A (C) or VRK2B (D) RNA expression against the VRK1 genetic dependency. Line denotes linear regression. CNS and PNS lineages are shown in red. For panel C: R^2=0.068 ; $p < 0.001$ (black) ; R^2=0.177 ; $p < 0.001$ (red). For panel D: R^2=0.021 ; $p < 0.001$ (black) ; R^2=0.15 ; $p < 0.001$ (red)

Supplementary Figure Legends continued

Supplementary Figure 4 VRK1 and VRK2 expression in GBM and NB tumors

A-B. UMAP plots from Cellligner-corrected *VRK2* (A) or *VRK1* (B) expression for RNA-sequencing on all available human tumors. Brain tumor (red box) and neuroblastoma (blue box) tumor lineage clusters are indicated with boxes. Right panels: violin plots showing Cellligner-corrected *VRK2* or *VRK1* expression for all tumor lineages (black) against brain cancers and NB (red).

C. Violet plots showing log2(TPM) RNA-sequencing data from the TREEHOUSE/TARGET dataset containing human neuroblastoma tumors. Tumors were separated on the basis of MYCN-amplification (top panel) or adrenergic/mesenchymal (bottom panel).

D. Immunoblot of basal protein levels of VRK1 and *VRK2* in a panel of pediatric H3.3K27M and H3 wild-type glioma cell lines.

*p < 0.05, **p < 0.001, ***p < 0.0001; Two-tailed, Student's T-test for all comparisons.

Supplementary Figure 5 VRK1 and VRK2 expression in normal tissues

A. Bisulfite sequencing showing cytosine methylation in a region covering 7 CpG dinucleotides in a CpG island found upstream of the transcriptional start site for both *VRK2A* and *VRK2B* isoforms (-300 to -244 nucleotides upstream of the TSS for *VRK2A*). Each circle denotes a CpG site within the region ; black denotes a methylated CpG and white denotes an unmethylated CpG.

B. Dot plot of *VRK2* RNA expression vs. gene promoter methylation (probe cg26093711 within CpG island) in TCGA GBM cohort (http://maplab.imppc.org/wanderer/). Spearman correlation = -0.817.

C. Violin plots showing Log2(TPM+1) mRNA expression for *VRK1* (top panel) and *VRK2* (bottom panel) across a panel of healthy tissues from GTEx (https://gtexportal.org/).

Supplementary Figure Legends continued

Supplementary Figure 6. Validation of paralog relationship of VRK1 and VRK2 through VRK2 depletion or over-expression

A. Immunoblot showing basal protein levels of VRK1 and VRK2 in a panel of GBM cell lines.

B. Immunoblot showing VRK2 protein expression following generation of isogenic cell line pairs in *VRK2*^{high} GBM cell lines through CRISPR KO of *VRK2*.

C. Incucyte time-lapse experiment of cell proliferation in SF172 cell line expressing 2x2 combinations of sgCtrl/Ctrl, sgCtrl/VRK1, sgVRK2/Ctrl, or sgVRK2/VRK1 guides. t = 0hrs is 7 days post-infection under antibiotic selection. Significance at each time point was determined by two-way ANOVA (treatment x time). * $p < 0.05$.

D. Immunoblot of exogenous wildtype or kinase-inactive VRK1^{WT}, VRK1^{K179E}, VRK2^{WT}, or VRK2^{K168E} following lentiviral transduction of LN443 and LNZ308 GBM cell lines.

E. Clonogenic assay of LN443 or LNZ308 GBM cell lines overexpressing VRK2^{WT} or kinase-inactive VRK2^{K168E} 3 weeks following lentiviral transduction with non-targeting guide or guides targeting *VRK1* (sgVRK1#1 and sgVRK1#2).

F. Clonogenic assay of the NB-1 neuroblastoma cell line overexpressing VRK2^{WT} or kinase-inactive VRK2^{K168E} 2 weeks following lentiviral transduction with sgCh2.2 control guide or sgVRK1 guide.

G. Immunoblot of protein expression levels of exogenous VRK1^{WT}, VRK1^{K179E}, VRK2^{WT}, or VRK2^{K168E} following lentiviral transduction of SU-DIPGXIIIP*Luci cells.

H. Effect of VRK2^{WT} or VRK2^{K168E} overexpression on SU-DIPGXIIIP*Luci cell viability following 10 days *VRK1* KO. (n=4; mean ± SD). Significance was determined by one-way ANOVA and Tukey's post-hoc test.

I. Effect of VRK1^{WT}/VRK1^{K179E} (left panel), VRK2A^{WT}/VRK2A^{K168E} (middle panel), or VRK2B^{WT}/VRK2B^{K168E} over-expression on Kelly NB cell line viability following *VRK1* KO (n=3; mean ± SD). Significance was determined by one-way ANOVA and Tukey's post-hoc test for the final day.

*$p < 0.05$, **$p < 0.001$, ***$p < 0.0001$, ns=not significant

Supplementary Figure 7. dTAG degrader system for ligand-induced VRK1 depletion

A. Left panel: Immunoblot validation of the dTAG-VRK1-dTAG degrader system in Kelly neuroblastoma cells. Exogenous dTAG-VRK1-dTAG was degraded in the presence of dTAG^{V}-1 (0.75 µM). Endogenous *VRK1* was independently targeted with CRISPR KO. sgNT is a non-targeting guide control. Right panel: Viability of dTAG-VRK1-Kelly cells following addition of either vehicle control or 0.75 µM dTAG^{V}-1. Significance at each time point was determined by two-way ANOVA (treatment x time). * $p < 0.05$

B. Incucyte time-lapse experiment of cell proliferation in dTAG-VRK1-LN443 cells following transduction of non-targeting guide (sgCtrl) or guide targeting *VRK1* (sgVRK1). t = 0hrs is 5 days post dTAG^{V}-1 (0.5 µM) addition. Significance at each time point was determined by two-way ANOVA (treatment x time). ** $p < 0.001$.

Supplementary Figure Legends continued

Supplementary Figure 8. Phospho-peptide quantification of LEM-domain containing proteins following acute VRK1 depletion

A. Change in phospho-peptide abundance at 4h and 8h following VRK1 degradation in dTAG-VRK1-NB-1 cells. Each point represents a separate phospho-peptide measured by quantitative phospho-proteomics. Highlighted are three nuclear membrane associated, LEM-domain containing proteins (TMPO, LEMD3, and EMD).

Supplementary Figure 9. Nuclear morphology changes following VRK1 and VRK2 depletion via decreased BAF phosphorylation

A. Nuclear membrane morphology in the SF172 GBM cell line following transduction with 2x2 combinations of sgCtrl/Ctrl, sgCtrl/VRK1, sgVRK2/Ctrl, sgVRK2/VRK1 guides. Nuclear membrane was visualized by immuno-fluorescent staining for LaminB1. White arrow points to nuclear bridge. Blue arrow points to micro-nuclei. Scale bar = 20μM.

B. Immunoblot of phosphorylated BAF (S4) and total BAF following 5 days doxycycline-induced expression of guide targeting *VRK1* in SU-DIPGXIIILuci DMG neurospheres. Representative of 2 independent experiments. pBAF and total BAF were probed in two separate blots of the same lysate.

C. Immunoblot following 3 days of doxycycline-induced expression of BAF^{WT}, BAF^{S4A}, BAF^{S4D} in LN443 GBM cells.

D. Time-lapse of live-cell experiment showing nuclear envelope morphology (GFP-tagged BAF) following VRK1 degradation in dTAG-VRK1-NB-1 NB cells undergoing mitosis ($dTAG^{V}$-1 = 0.5μM). Scale bar = 20μM.

Supplementary Figure 10. *In vivo* validation of VRK1 dependency in neuroblastoma xenografts

A. Population doubling assay in Kelly neuroblastoma cells expressing a doxycycline-inducible sgRNA targeting either Ctrl or one of two guides targeting VRK1 and treated for 11 days with vehicle or 0.5 μg / mL doxycycline (n=3; mean ± SD). Significance at each time point was determined by one-way ANOVA and Tukey's post-hoc test on each day. * p < 0.05, **p < 0.001, ***p < 0.0001

B. Western blot showing VRK1 expression in cell lines and treatments shown in panel A.

C. Volume measurements over time of flank xenografts of the Kelly GBM cell line transduced with Cas9, and Doxycycline-inducible guide vector against cutting control (sgCh2-2, N=6 tumors) or VRK1 (sgVRK1, N=8 tumors). When the tumors reached a pre-specified size (~50 mm^3), the mice were switched to doxycycline-containing chow (625pp). * represents treatment with Doxycycline.

D. Kaplan-Meier survival curves showing overall survival for mice in panel C. Control vs. doxycycline. Significance as determined by log-rank test. p=0.14.
